# High yield purification of an isoleucine zipper-modified CD95 ligand for efficient cell apoptosis initiation and with biotin or DNA-oligomer binding domain to probe ligand functionalization effects

**DOI:** 10.1186/s12896-025-00986-2

**Published:** 2025-07-01

**Authors:** Xiaoyue Shang, Nina Bartels, Johann Moritz Weck, Sabine Suppmann, Jérôme Basquin, Gajen Thaventhiran, Amelie Heuer-Jungemann, Cornelia Monzel

**Affiliations:** 1 Present Address: 2nd Institute of Physics, University of Stuttgart, Pfaffenwaldring 57, 70569 Stuttgart, Germany; 2 Experimental Medical Physics, Heinrich Heine University Düsseldorf, Universitätsstraße 1, 40225 Düsseldorf, Germany; 3 Max Planck Institute of Biochemistry, Am Klopferspitz 18, 82152 Martinsried, Germany; 4 Fraunhofer Institute for Translational Medicine and Pharmacology ITMP, Nonnenwald 2, 82477 Penzberg, Germany; 5 Present Address: Hybrid Bionanosystems, TU Dortmund, Otto-Hahn-Str. 4a, 44227 Dortmund, Germany

**Keywords:** CD95 ligand, Isoleucine zipper, Mammalian expression, Cell factory, Site-specific modification, DNA origami, Cell apoptosis

## Abstract

**Background:**

Cluster of differentiation 95 (CD95/Fas/Apo1) as part of the Tumor-necrosis factor (TNF) receptor family is a prototypic trigger of the ‘extrinsic’ apoptotic pathway and its activation by the trimeric ligand CD95L is of high interest. However, CD95L, when presented in solution, exhibits a low efficiency to induce apoptosis signaling in human cells.

**Results:**

Here, we design a recombinant CD95L exhibiting an isoleucine zipper (IZ) motif at the N-terminus for stabilization of the trimerized CD95L and demonstrate its high apoptosis initiation efficiency. This efficiency is further enhanced by antibody-mediated crosslinking of IZ-CD95L.A cysteine amino acid fused behind the IZ is used as a versatile coupling site for bionanotechnological applications or for the development of biomedical assays. A fast, cheap, and efficient production of CD95L *via* the HEK293T secretory expression system is presented, along with CD95L affinity purification and functionalization. We verified the biological activity of the purified protein and identified a stabilized trimeric CD95L structure as the most potent inducer of apoptosis signaling.

**Conclusions:**

The workflow and the findings reported here will streamline a wide array of future low- or high-throughput TNF-ligand screens, and their modification towards improving apoptosis induction efficiency and, potentially, anticancer therapy.

**Supplementary Information:**

The online version contains supplementary material available at 10.1186/s12896-025-00986-2.

## Background

CD95 ligand (CD95L, Fas ligand, CD178, or TNFSF6) is a 40 kDa type II transmembrane protein belonging to the tumor necrosis factor (TNF) superfamily [1]. It is expressed on multiple immune cells such as cytotoxic T lymphocytes and monocytes [2].

Over the last decades, CD95L and other death-inducing ligands of the TNF super-family, such as TNF and TNF related apoptosis inducing ligand (TRAIL), have garnered significant attention due to their potential to trigger the programmed cell death of apoptosis [3] and their further application in cancer therapy [4]. However, next to their active role in cell fate determination, these ligands turned out to be involved in other cellular responses/outcomes. For example, TNF, the founding member of this family, binds to TNFR1 or TNFR2 and can either trigger pro apoptosis (TNFR1) or pro inflammation (TNFR2) pathways. Also, due to the widespread expression of TNFR1 across various cell lines, the selectivity of TNF-based cancer therapy is limited. TRAIL, on the other hand, binds to four receptors, DR4, DR5, DcR2 and OPG, which results in a complex signaling landscape. In case of CD95L, heterogeneous signaling outcomes have also been reported: for a long time, CD95L was known to trigger the programmed cell death of apoptosis in vitro [3].

**Fig. 1:**
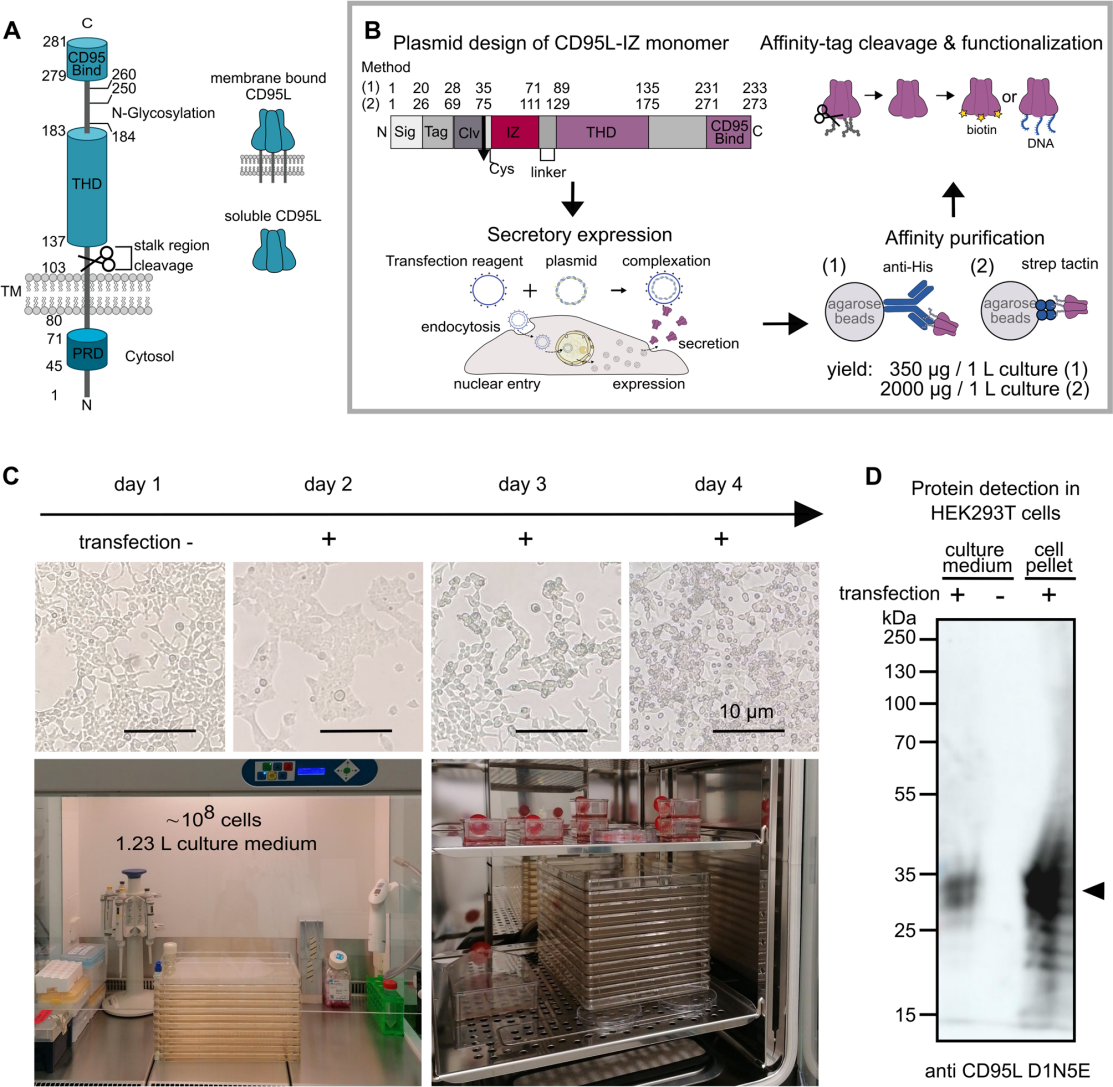
CD95L structure and experimental design. **A** CD95L can exist either in a membrane-bound or in a soluble form by protease cleavage. Key domains include the CD95 binding domain (CD95 Bind), the TNF homology domain (THD), and the proline-rich domain (PRD). **B** Workflow of the IZ-CD95L construct design, secretory expression, affinity purification, and modification. The recombinant protein consists of a signaling peptide (Sig), an affinity tag (Tag), a cleavage site (Clv), a Cysteine (Cys), an isoleucine zipper domain (IZ) to CD95L extracellular domain. In method 1 transient IZ-CD95L expression was used. Here, a His-tag was used as Tag and a TEV protease sequence as Clv. Affinity purification was done by anti-His-tag antibody coupled agarose beads. In method 2 a cell line stably expressing IZ-CD95L was used. Here, a Twin-Strep-Tag was used as Tag and a PreScission cleavage site as Clv. Affinity purification was done by Strep-TactinXT4Flow beads. **C** Upper panel: examination of cell morphology before and up to three days after transfection. Lower panel: photos of cell culture and transfection using a 10-layer cell factory in a cell culture hood (left) or incubator (right). A T175 cell culture flask was seeded with the same cell density as in a 10-layer cell factory to check the cell condition and confluency. **D** IZ-CD95L protein expression was verified by western blot of culture medium by anti-CD95L (D1N5E) antibody

More recently,accumulating evidence indicates another role of CD95L/CD95 in alternative nonapoptotic signaling pathways leading to proliferation or migration, and consequently, tumorigenesis [5, 6]. While activation of the proliferation pathway was suggested to involve additional interactions on the intracellular site, such as tyrosine phosphorylation of the intracellular CD95 death domain or of caspase-8, both by src-family kinases (SFKs) [7–10], the mechanisms triggering the proliferation pathway are still little understood. Finally, in vivo studies reported severe tissue damage induced by the CD95L system [11] and intravenous administration of CD95L resulted in lethal effects in mice [12]. Such acute tissue damage has limited the CD95/CD95L pathway implication in cancer therapy, albeit efforts are made to develop CD95L inhibitors. Still, CD95L is known to exhibit only a single interaction with the receptor CD95 (Fas, Apo-1), wherefore it is chosen here as a well-controllable system to study effects of CD95/CD95L interactions on the signaling in in vitro experiments, where cell apoptosis is primarily triggered. Interestingly, the efficiency of inducing the apoptosis pathway appears to also largely depend on the presentation and oligomerization of CD95L [1]: for example, CD95L can exist in a membrane-anchored (mCD95L) as well as in a soluble form (sCD95L). In case of the soluble form, CD95L is cleaved by matrix metalloproteases (MMPs) at its stalk region [13] (see Fig. 1A). Several studies showed that this soluble form of CD95L is highly inefficient in inducing apoptosis in comparison to mCD95L and may instead trigger pro-inflammatory signaling pathways [14–18]. This reduced cytotoxicity is also common among other cleaved TNFSF ligand and it has been speculated, whether this results from a change in the secondary molecular structure.Other studies showed, that sCD95L gets accumulated upon binding to CD95 and, once it reaches a sufficient aggregation level, allows the initiation of the cell death program [19, 20]. Similarly, a recombinant form of two trimers was efficiently inducing cell death [21], supporting that the extent to which CD95L is multimerized plays a pivotal role in determining whether cell apoptosis signaling is induced or not.

CD95L has a long cytoplasmic domain with its N-terminus, a transmembrane (TM) domain, a stalk region, a TNF homology domain (THD) that mediates homotrimerization [1], and a CD95 binding domain at its C-terminus (see Fig. 1A). Both, the membrane-anchored and soluble forms of CD95L, form homotrimers [14]. However, the studies mentioned above suggest that the binding of naturally occuring sCD95L to CD95 exhibits a reduced efficiency to induce cell death [16], putatively due to a missing secondary stabilization of the trimer at the stalk region compared to the membrane-bound CD95L. Indeed, several structural modifications or intermolecular crosslinking approaches have been tested to probe and improve the sCD95L apoptosis efficiency (see discussion for examples). In this work, we show how trimerization by an isoleucine zipper (IZ) domain can increase the cytotoxicity of soluble CD95L without additional cross linkers and with the goal to obtain new insights into the mechanism of apoptosis signal induction.

We use IZ, a variant of the leucine zipper (LZ) as a self-crosslinking domain of CD95L, as it is a dimerization motif/coiled-coil motif found in eukaryotic transcription factors [22]. Each monomer forms an alpha helix with a periodic repetition of leucine residues at every seventh position. These motifs are characterized by a heptad repeat containing hydrophobic residues at the first (a) and fourth (d) positions, facilitating hydrophobic interactions and dimerization [23]. The variant of the wild-type GCN4 leucine zipper used, is the isoleucine zipper (IZ), which adopts parallel trimer structures with isoleucine substitutions at all a and d positions [24, 25]. The isoleucine zipper is a relatively small and straightforward motif, which can be advantageous when minimizing the overall size of the fusion protein. This facilitates easier expression, folding, and purification of the protein compared to larger domains like the T4 foldon or tenascin-C.

To tailor CD95L constructs towards their efficient apoptosis induction requires their genetic modification and the appropriate expression system to obtain the protein cost-effectively and at a high yield. Soluble CD95L expression and purification have been widely studied in both eukaryotic (*Dictyostelium discoideum*, COS cells) [26–29] and prokaryotic expression systems, such as *Escherichia coli* (*E.coli*) [30]. The bacterial *E.coli* expression system has been the most popular method for recombinant protein expression and purification, due to the benefits of cost-efficiency, time-saving, and high yield at the milligram scale [31]. However, post-translational modifications such as disulfide-bond formation and glycosylation are very different in prokaryotes compared to mammalian cells [32]. Previous studies have demonstrated that mutations at CD95L glycosylation sites do not significantly affect self-aggregation or receptor binding [33]. However, these mutations reduce the CD95L expression level in transfectants [34], indicating a positive relation between glycosylation and protein maturation and stability. Glycans can also protect proteins from proteolytic degradation by masking protease sites, thereby prolonging the half-life of glycoprotein at the cell surface [35].

While low yield has always been the cutting limit of human protein expression and purification using an adherent cell line, the most appropriate host to maintain the native folding environment and modifications is the human cell line HEK293 (human embryonic kidney 293). In the past, also the adapted suspension cell line HEK293F as a fast-growing variant has been established [36]. Another variant, which was used in this work, is the HEK293T cell line, a highly transfectable derivative of the HEK293 cell line, due to the incorporation of a temperature-sensitive mutant of SV40 large T-antigen (tsA1609). This cell line maintains a high copy number of transfected plasmid DNAs which carry the SV40 origin of replication [37].

We here designed a CD95L fused with an isoleucine zipper domain, which enhances apoptosis efficiency without the need for additional crosslinking. We present an efficient production of IZ-CD95L using the mammalian HEK293T cell line in large cell culture vessel, the cell factory. The ligands were expressed by firstly transient transfection and purified by anti-His affinity purification (method 1). We then used the findings from method 1 to develop an alternative strategy based on a stable cell line expression and purification via a strep-tactin resin to increase the yield and to simplify the cell cultivation process (method 2). In both cases we obtained IZ-CD95L at high purity and yield (see Methods for details). We characterize the IZ-CD95L oligomeric state and demonstrate its functionality to create bionanotechnological or biomedical assays with a high efficiency to induce apoptosis in cancer cells.

## Methods

Most of the presented data have been acquired using IZ-CD95L from method 1. In addition, a HEK293T cell line was created stably expressing IZ-CD95L. Here, we describe and compare the handling in both cases along with the expression efficiencies. First the methods for transient transfections are described followed by methods for the stable cell line expression.

### IZ-CD95L plasmid design for transient expression

For the plasmid IL2pept-His8-TEV-Cys-IZ-CD95L, the extracellular domain of CD95L consisting of amino acids 137–281, was fused at its N-terminus with an IZ domain, which stabilizes the trimerization of CD95L. The stalk region (103–137) has been reported to enhance the efficacy of both murine CD95L [38] and human CD95L [39] compared with sCD95L. However, fusion with a FLAG tag in conjunction with the stalk region (FLAG-stalk-CD95L) has been shown to reduce cytotoxic activity [39], whereas IZ-stalk-CD95L exhibited increased cytotoxicity. Given the potential steric hindrance of the stalk region on the IZ domain we opted for to using CD95L without stalk region. One extra cysteine was added after the IZ for further functionalization (biotinylation or DNA conjugation). Next to the cysteine, a TEV protease cleavage site ENLYFQ was incorporated along with 8 histidines, to allow for His-tag purification. To allow protein secretion, we used the interleukin 2 (IL2) signal peptide MRRMQLLLLIALSLALVTNS as the N-terminal sequence, which has been extensively studied and widely used in human protein production in industry and academia [40–42]. Specifically, we used a mutant version of the IL-2 signal peptide (IL2pept) with modifications in the basic and hydrophobic domains, which resulted in a three-fold increase in protein secretion levels compared to the wild type [43]. The complete amino acid sequence of the insert reads MRRMQLLLLIALSLALVTNSHHHHHHHHENLYFQGCGDRMKQIEDKIEEILSKIYHIENEIARIKKLIGERTSGGSGGTGGSGGTGGSPPEKKELRKVAHLTGKSNSRSMPLEWEDTYGIVLLSGVKYKKGGLVINETGLYFVYSKVYFRGQSCNNLPLSHKVYMRNSKYPQDLVMMEGKMMSYCTTGQMWARSSYLGAVFNLTSADHLYVNVSELSLVNFEESQTFFGLYKL*. It has a theoretical isoelectric point and molecular weight pI / M.W. of: 9.10 / 26,312, calculated by Expasy (https://web.expasy.org/compute_pi/). The corresponding DNA was inserted into a plasmid with a pcDNA3.1(-) backbone with a 5‘ restriction site EcoRI and 3‘ restriction site BamHI. The plasmid was ordered from BioCat (BioCat GmbH, Heidelberg, Germany) with codon optimization.

### His-IZ-CD95L plasmid amplification

For large-scale His-IZ-CD95L expression, a milligram amount of plasmid DNA was required. The plasmid was purified using a NucleoBond^®^ Xtra Maxiprep kit (Macherey-Nagel, Düren, Germany). The plasmid was transformed into $\:\text{D}\text{H}5\alpha\:$ competent cells (Thermo Fisher Scientific, Waltham, Massachusettes, USA) *via* heat shock and selected on LB/ ampicillin (lysogeny broth) agar plates overnight at 37 °C. Thereafter, a single colony was picked for a preculture in 5 ml 2x YT/ ampicillin (yeast extract tryptone) medium for 5 h. 4 × 500 ml main culture of were grown at 37 °C and 200 rpm o/n. From each 500 ml culture, 2 ml of purified plasmid at a concentration of 1.4–1.9 µg/µl was obtained and verified by sequencing. Of note, for continuous IZ-CD95L production, the establishment of a stable cell line expressing IZ-CD95L is recommended, since no large amount of plasmid DNA is required, and cells can be sorted for those exhibiting a high transfection efficiency. The method of stable cell line establishment and ligand purification is described below in the subsection.

### Cell culture

Hela wild type (Hela WT), human embryonic kidney 293T (HEK293T), and Michigan Cancer Foundation-7 (MCF7) cells are ordered (both from ATCC^®^ CCL-2™, ATCC, Manassas, VA, USA). Hela CD95 knock out (Hela CD95^KO^) stable cell line was generated using clustered regularly interspaced short palindromic repeats (CRISPR/Cas9) [44], the guide RNA was CATCTGGACCCTCCTACCTC [45]. All cell lines were cultured in Dulbecco’s modified eagle medium (DMEM) + GlutaMAX™ (Gibco, Life Technologies Inc., Carlsbad, CA, USA) containing 10% fetalbovine serum FBS (Gibco) and 1% penicllin/streptomycin (P/S) solution (Sigma-Aldrich, Merck KGaA, Darmstadt, Germany) at 5% (v/v)CO_2_ in a humidified incubator. Cells were maintained in T25 flasks (Sarstedt AG & Co. KG, Nümbrecht, Germany) and were passaged every 2–3 days at a confluency level of around 60–80%.

### Large-scale expression of His-IZ-CD95L using cell factory

Before large-scale cell culture and transfection, a small-scale test expression and verification was performed in a T75 flask. The large-scale production of His-IZ-CD95L was performed using a 10-layer cell culture vessel (Nunc™ EasyFill™ Cell Factory™ Systems, Thermo Fisher Scientific). Before using the cell factory, the number of cells needed to be scaled up to $\:{\sim\:10}^{8}$ for sufficient cell density. To this end, cells were first grown in a T25 flask, then transferred into a T175 flask and grown nearly to confluency. After 3 days, the up to 2.3 x $\:{10}^{7}$ cells which can be grown in a T175 flask, were distributed among seven T175 flasks and again grown to near confluency (80–90%). Before the transfer, 1.23 L of DMEM complete (10% FBS + 1% P/S) was filled into the 10-layer cell factory under sterile conditions and all cells were evenly distributed among the layers. Since it is difficult to monitor the cell growth in the 10-layer cell factory, another T175 flask was prepared in parallel at the same cell surface coverage. After 3 days of growth, when cells reached a confluency of 70–80%, the DMEM complete medium in the cell factory was carefully replaced with the Opti-MEM complete (10% FBS + 1% P/S) medium. Care should be taken to keep the inlet of the flasks dry to reduce the risk of contamination. For transfection, 1.28 mg of plasmid DNA was diluted into 60 ml Opti-MEM plain, and 3.84 mg of PEI was diluted into 60 ml Opti-MEM plain (DNA/PEI mass ratio of 1:3), the solutions were mixed and the 120 ml transfection mixture incubated at r.t. for 30 min. Afterwards, the mixture was added into a 1.23 L fresh Opti-MEM complete medium. Cell morphology and sterile conditions were checked every day after transfection. 72 h after transfection started, 1.35 L of cell culture medium was harvested. On average, this yielded an amount of 350 µg purified protein per 1 L culture medium.

### His-tag affinity purification

After cell culture medium collection, the cell debris was removed by centrifugation at 1300 g for 5 min. Large particles were removed by filtration with a 0.2 μm cut-off filter (Filtropur S 0.2, Sarstedt, Nümbrecht, Germany). The cell culture medium was concentrated using Amicon^®^ Ultra-15 3 K centrifugal filters (Merck Millipore Ltd., Burlington, MA, USA) into a final volume of 80 ml to increase the ligand concentration in medium and the binding efficiency to affinity resin in the following step. For purification of His-IZ-CD95L, 2 ml of anti-His-tag affinity resin (GenScript Biotech, Piscataway, NJ, USA) was added into the concentrated culture medium and incubated at 4 °C overnight with rotation. Thereafter, two empty polypropylene 1 ml columns (QIAGEN GmbH, Hilden, Germany) were equilibrated with 5 ml of 20% ethanol, 5 ml of ultrapure water, and 5 ml TBS buffer (Tris-buffered saline, 50 mM Tris·HCl, 150 mM NaCl, pH 7.4). After the overnight incubation, the resin-culture medium mixture was loaded on both columns (each column volume: 40 ml). The loaded column was washed with 30 ml TBS buffer to rigorously remove non-specifically bound protein. Considering the theoretical pI of CD95L of 8.8 (for the His-tagged construct but without the cleaved signaling peptide after secretion), the His-IZ-CD95L was eluted following the GenScript manual using alkaline elution buffer (0.1 M Tris·HCl, 0.5 M NaCl, pH 12.0) with 12 ml total elution volume. After additional concentrating the 12 ml elution using Amicon^®^ Ultra-15 3 K, the His-IZ-CD95 concentration was determined by Pierce^®^ BCA Protein Assay Kit (BCA assay, Thermo Fisher Scientific). This yielded a protein concentration of 0.409 mg/ml. The protein size and purity were checked by SDS-PAGE using 10% precast gel (Mini-PROTEAN^®^ TGX™, Bio-Rad). Here, 1 µg purified protein was loaded into 25 µl Laemmli sample buffer containing 50 mM TCEP·HCl and heated at 95 °C for 5 min followed by 180 V electrophoresis for 1 h. The gel was stained with SimplyBlue™ Safe Stain (Thermo Fisher Scientific).

### Size exclusion chromatography

Size exclusion chromatography was performed using the Superdex 75™ 10/300 GL (Cytiva) column with the ÄKTA prime plus (Cytiva) (Fig. 3A) or ENrich TM Sects. 70 10 × 300 column (Biorad) using NGC chromatography system (Biorad) (Fig. 3B, C). The column and system were equilibrated with 2 column volumes (CV) of 20% ethanol, 2 CV of water, and 2 CV of phosphate-buffered saline (PBS) buffer at pH 7.4. Either 350 µl of purified His-IZ-CD95L (0.409 mg/ml) or a standard protein mix 15–600 kDa at 5.5 mg/ml (Sigma-Aldrich) or 200 µl 3.5 mg/ml (Aglient, AdvanceBio SEC Standards) was loaded and eluted at 0.3 ml/min flow rate with 0.3 ml fraction size in PBS buffer. Collected protein fractions were analyzed by SDS-PAGE (Figure S2 D-E). Elution volume of 670 kDa protein was used as void volume, and the rest 4 proteins were used for standard curve. Elution fractions of biotin-IZ-CD95L were concentrated and tested for their cytotoxicity by CTB assay.

### Western blot

Western blot was performed to verify His-IZ-CD95L in culture medium. The medium with or without transfection after 72 h was concentrated by 14x and 11x using 3 K centrifuge filters (Sigma-Aldrich). For transfection +: 6 µl medium + 2 µl of 5x sample buffer / TCEP·HCl + 2 µl milliQ was heated (95 °C, 5 min) and loaded. For transfection -: 8 µl medium + 2 µl of 5x sample buffer / TCEP·HCl + 2 µl milliQ was heated and loaded. Proteins were first separated by vertical electrophoresis (Bio-Rad) with tris-glycine-SDS running buffer (Thermo Fischer Scientific) at 130–200 V. Thereafter, for western blot, a ROTI^®^PVDF membrane (Carl-Roth GmbH & Co. KG, Karlsruhe, Germany) was activated with 100% methanol for 1 min, distilled water for 2 min and blotting buffer (25 mM Tris, 192 mM Glycine, pH 8.3, 20% methanol) for 5 min. The membrane was sandwiched by two pieces of thick filter paper (diameter 110 mm, prewet in blotting buffer for 5 min) and the protein was transferred using a semi-dry system (Bio-Rad) at 25 V 2.5 A for 20 min. The membrane was blocked with 5% albumin fraction V BSA in TBST buffer (tris-buffered saline, 0.1% tween-20) at r.t. for 1 h and probed with primary antibody at 4 °C overnight with 1:500 anti-His-tag antibody (BioLegend, J099B12) and 1:1000 anti-CD95L antibody (Cell Signaling, D1N5E). The next day, after rigorous washing 4 × 10 min each with TBST, the membrane was treated with goat anti-mouse (1:1000) or goat anti-rabbit (1:1000) secondary antibody coupled with horseradish peroxidase (HRP) (Cell Signaling Technology, Danvers, MA, USA) at r.t. for 60 min. Chemiluminescence detection was achieved by treating with Westar eta c ultra 2.0 substrate (Cyanagen Srl, BO, Italy) for 10–30 s. Images were taken using the Amersham™ Imager AI680 (Cytiva).

### His-tag cleavage

200 µg of freshly eluted His-IZ-CD95L was reduced with 5 mM dithiothreitol (DTT) and incubated with 13 µg (1:15 w/w) TEV protease (Sigma-Aldrich) at 4 °C overnight. The next day, 50 µl of HisPur™ Ni-NTA magnetic beads (Thermo Fisher Scientific) were added in a 1.5 ml Eppendorf tube and washed with first 200 µl and then 500 µl of PBS buffer by mixing followed by collecting the beads with a magnetic stand and discarding the supernatant. Thereafter, 200 µg of the protein mixture was added and mixed by pipetting for 10 s. For coupling the His-IZ-CD95L to the beads, the mixture was rotated for 30 min at r.t. The beads were magnetically trapped and the supernatant containing IZ-CD95L without any His-tag was collected and checked by western blot. 60 ng of His-IZ-CD95L and IZ-CD95L after His-tag cleavage was loaded first on a 10% SDS-PAGE followed by a western blot probed with anti-His-tag antibody 1:1000 (BioLegend, San Diego, CA, USA). After His-tag cleavage, DTT must be removed due to the competition of coupling sites with maleimide. This was achieved by buffer exchange with PBS buffer using Amicon^®^ Ultra-0.5 3 K centrifugal filters (Merck Millipore Ltd.).

### Formation of stable cell line and protein purification

HEK293T (ATCC^®^ CRL-3216™) cell pools stably expressing Twin-Strep-tagged IZ-CD95L fusion protein were generated using piggyBac transposase as described previously [46]. This expression system uses three components: the tetracycline-inducible expression vector PB-T-PAF, the helper plasmid PB-RN for transactivation of the Tet promotor and the plasmid expressing hyperactive piggyBac transposase under the control of a CMV promotor. To ensure efficient secretion, the human VEGF leader sequence (MNFLLSWVHWSLALLLYLHHAKWSQA) was used followed by a N-terminal Twin-Strep-Tag in combination with a PreScission cleavage site LEVLFQ/GP for tag removal. The complete amino acid sequence of the insert reads MNFLLSWVHWSLALLLYLHHAKWSQAAPMAEGGGQNSAWSHPQFEKGGGSGGGSGGSAWSHPQFEKTAGLEVLFQGPGCGDRMKQIEDKIEEILSKIYHIENEIARIKKLIGERTSGGSGGTGGSGGTGGSPPEKKELRKVAHLTGKSNSRSMPLEWEDTYGIVLLSGVKYKKGGLVINETGLYFVYSKVYFRGQSCNNLPLSHKVYMRNSKYPQDLVMMEGKMMSYCTTGQMWARSSYLGAVFNLTSADHLYVNVSELSLVNFEESQTFFGLYKL*.

Briefly, PB-T-PAF-IZ-CD95L under the control of an inducible Tet-Promotor was co-transfected with PB-RN and pCMV-HypBase, selected with puromycin for three weeks, and further expanded without selection. For protein production, stably IZ-CD95L expressing HEK293T cell pools were grown to a density of 1 × 10^6^ cells / mL in HEK FreeStyle293 medium (Gibco), gene expression was induced using 1 µg/mL doxycycline and culture supernatant was harvested three days post-transfection.

For IZ-CD95L purification (see Supplementary Fig. 7), 2 L prefiltered culture supernatant was treated with 4 mL BioLock (IBA LifeSciences, Göttingen) for 15 min on ice to block medium biotin content and subsequently loaded on 2 mL Strep-TactinXT4Flow beads (IBA LifeSciences, Göttingen). Beads were washed with 20 mL PBS and protein was eluted in PBS + 50 mM biotin. Eluted fractions were pooled for overnight in-solution cleavage at 4 °C with HRV-3 C-Protease (His-3 C; Sigma-Aldrich). His-3 C was depleted on the His-Select Affinity matrix (Sigma-Aldrich) prior to Size Exclusion Chromatography on a HiLoad 16/60 Superdex200 column (Cytiva, Germany) in PBS. Major peak fractions were analyzed by SDS-PAGE (see Supplementary Fig. 7), pooled and concentrated using Vivaspin Turbo at 10 kDa cutoff (Sartorius Stedim Biotech, Göttingen) to a final concentration of 0.4 mg/mL. The final yield was 4 mg / 2 L culture.

### Site-specific labelling (biotinylation or DNA conjugation)

100 µg of protein in PBS was reduced freshly with 2 mM TCEP·HCl at 4 °C for 30 min. For biotinylation, 10x molar excess of biotin-C5-maleimide (Sigma-Aldrich) in comparison to the protein was incubated with IZ-CD95L at 4 °C o/n (i.e. 2 µl of 10 mg/ml biotin-C5-maleimide was added). The next day, extra biotin was removed using NAP™-5 column (Cytiva). Here, the NAP-5 column was washed with 10 ml PBS buffer and then loaded with 120 µl of protein/biotin. After equilibration of the column with 0.38 ml PBS, the protein was eluted with 0.53 ml PBS. The final concentration of the biotinylated protein was 0.189 mg/ml in 550 µl. Biotinylation was checked *via* western blot: 300 ng of IZ-CD95L and biotin-IZ-CD95L was analyzed on 10% SDS-PAGE followed by western blot and probed first by AF488-streptavidin (AAT Bioquest, Pleasanton, CA, USA) binding at 30 µg/ml overnight at 4 °C. Equal loading of IZ-CD95L/ biotin-IZ-CD95L was qualitatively checked by re-probing the same membrane with anti-CD95L antibody (Abnova, Taiwan). For DNA conjugation, potential disulfide bridges after His-tag cleavage were reduced by addition of TCEP to a final concentration of 2 mM and incubation for 30 min at 4 °C. Afterwards DNA-maleimide (biomers.net GmbH) was added in 5x excess over the IZ-CD95L trimer and incubated overnight at 4 °C. The ssDNA-IZ-CD95L construct was then purified with Amicon centrifugal filters (Merck Millipore, 30 kDa cutoff, cat.no.: UFC5030), with ten PBS washing steps at 8000 rcf and 4 °C for 4 min each.

### Native PAGE

To see the band shift of IZ-CD95L after CD95 binding under native conditions, we used clear native PAGE (CN-PAGE) and blue native PAGE (BN-PAGE). Here, we considered IZ-CD95L with a theoretical isoelectric point (pI) of 8.8 as a basic protein and CD95 with a pI of 6.8 as an acidic protein at physiological pH. For CN PAGE, a hand-cast native gel without SDS (7.5% acrylamide, 0.1% ammonium persulfate (APS), 0.1% tetramethylethylenediamine (TEMED), 375 mM Tris, pH 9.4) was freshly prepared. Protein samples of 100 ng CD95-His incubated with 100 ng biotin-IZ-CD95L at 37 °C for 1 h, or 100 ng CD95-His alone (Sino Biological, Beijing, China) were loaded with 10% glycerol into 10 µl loading volume. Electrophoresis was performed at 4 °C 200 V for 0.5 h with running buffer (50 mM Tris, 192 mM Glycine, pH 9.14). Afterwards, the proteins from the native gel were transferred to the PVDF membrane and probed with an anti-His-tag antibody (BioLegend). For BN-PAGE, protein samples of 100 ng CD95-His alone or with 10x molar excess of IZ-CD95L or FLAG-CD95L (Enzo) were loaded into a precast gel (Biorad) with 50% glycerol and 5% Coomassie blue G-250 with a final volume of 10 µl. The electrophoresis was performed with 50 V, 75 V, 100 V gradient voltage each for 30 min at 4 °C with anode running buffer (25 mM Tris/ 192 mM glycine, pH 8.3) and cathode running buffer (25 mM Tris/ 192 mM glycine/ 0.02% G-250, pH 8.3). Afterwards, proteins from the native gel were transferred to the PVDF membrane and probed with anti-CD95 antibody (Miltenyi Biotec, clone DX2).

### Droplet assay

8 µl of 0.1 mg/ml biotinylated protein biotin-IZ-CD95L was mixed with 3.1 µl of ATTO594 NHS-ester (stock concentration of 18 µM, ATTO-TEC GmbH, Siegen, Germany) and 11.1 µl of the reaction mix was incubated at 4 °C overnight. The next day, 4.3 µl of the above reaction mix was taken and diluted in Phosphate-buffered saline (PBS) at pH 7.4 at a final protein concentration of 10 µg/ml in 31 µl. A drop of the protein solution was placed in one well of an 8-well chamber (Sarstedt) and incubated at r.t. for 1 h. The droplet was pipetted out and shortly air dried to ensure negligible free protein in the solution. Thereafter, the whole chamber surface was covered with 400 µl of 10 µg/ml BSA (ITW Reagents) and incubated at r.t. for 1 h. The BSA droplet was removed and the chamber was washed by 3 × 800 µl PBST buffer (phosphate-buffered saline, 0.1% tween-20 at pH 7.4) followed by incubation with 400 µl of 10 µg/ml AF488-streptavidin (AAT Bioquest) at r.t. for 1 h. The chamber was washed with 5 × 800 µl PBST afterwards. The sample was imaged with an IX83 inverted microscope (Olympus Europa SE & CO. KG, Hamburg, Germany) using a 20x air objective (NA 0.85, UPLSAP 20x O, Olympus). By fluorescence imaging using suitable filters in the 594 nm and 488 nm excitation channels, colocalization of the biotinylated protein and streptavidin was checked to verify successful protein biotinylation.

### CellTiter-blue^®^ viability assay

Cell viability assay was performed using CellTiter-blue^®^ (CTB) reagent (Promega) according to the manufacturer’s protocol. In detail, Hela WT and MCF7 cells were seeded into 96-well plates (Sarstedt) in triplicates, with densities of 25,000 cells per well. 24 h after seeding, cells were incubated with conditioned X-IZ-CD95L, FLAG-CD95L (Enzo) or His-CD95L (Prospec) in 200 µl DMEM at 37 °C for 16 h. 0.1% Triton X-100 was incubated on cells for 30 min as the negative control (0% cell viability), DMEM medium was incubated on cells for the positive control (100% cell viability). Next, cells were washed with DPBS followed by 200 µl 1:10 dilution of CTB stock in DMEM for 4 h at 37 °C. Well plate was analysed using Infinite M Plex plate reader (Tecan) with excitation at 560 nm and emission at 590 nm. Data was background corrected using negative control and normalized using positive control and averaged from three replicates.

### ELISA of CD95 and biotin-IZ-CD95L binding

CD95-His (Sino Biological) was coated on a 96-well plate at 2 µg/ml concentration in 50 µl PBS buffer overnight at 4 °C. Each well was washed 3 times with 180 µl PBS-0.05% tween-20 after each incubation step. The next day, wells were washed 3 times and blocked with 180 µl 2% BSA in PBS − 0.05% tween-20 for 1 h at r.t. biotin-IZ-CD95L or FLAG-CD95L (Enzo Life Sciences Inc., Lörrach, Germany) was diluted from 0.1 nM to 100 nM in 0.5% BSA/PBST and incubated with CD95-His at r.t. for 2 h followed by washing. Primary anti-CD95L antibody (Abnova) was diluted into 10 µg/ml in 50 µl 0.5% BSA/PBST and incubated overnight at 4 °C. The next day, wells were incubated with goat anti-mouse HRP linked secondary antibody (Cell Signaling) with 1:1000 dilution in PBST for 1 h at r.t., each well received 50 µl. Afterwards, 100 µl 1-StepTM slow TMB ELISA substrate (Thermo Fisher Scientific) was added into each well for 30 min for color development. Absorbance was measured at 652 nm with a Tecan Infinite 200PRO (Tecan Life Sciences). Absorbance values were corrected for the background signal and normalized by dividing with the maximum value. Data points were fitted using Matlab (R2018a, The MathWorks, Inc., CA, USA) with a 4-parameter logistic model.

### Apoptosis dynamics assays

Apoptosis dynamics assays were performed to demonstrate the killing efficiency of X-IZ-CD95L (His-IZ-CD95L /IZ-CD95L/biotin-IZ-CD95L) and its inhibition by CD95-His (Sino Biological) or enhancement by anti-His-tag antibody (BioLegend). FLAG-CD95L (Enzo) was assayed for a comparison of the apoptosis induction rate with X-IZ-CD95L. Hela WT cells were seeded in the wells of an 8-well glass slide with thickness No.1.5 (Sarstedt) at least 24 h before the experiment and the DMEM complete culture medium was replaced with pre-warmed 200 µl Leibovitz‘s L-15 medium without phenol red (Gibco) supplemented with 10% FBS (Gibco) + 1% P/S (Sigma-Aldrich) just before X-IZ-CD95L addition. X-IZ-CD95L (i) alone or (ii) incubated with CD95-His receptor or with (iii) anti-His-tag antibody (at r.t. for 30 min) were added to the 8-well plate with a final volume of 300 µl in each well at the desired concentration. As a general control, cells on one well were incubated with L-15 complete medium without ligand, where no apoptosis of cells was observed. As another control to exclude unspecific killing, the Hela CD95^KO^ cell line was incubated with the secreted ligand in L-15 medium after 3 days of the expression. Again, no apoptosis was observed. The 8-well chamber was mounted on an IX83 inverted microscope (Olympus) using a 20x air objective (NA 0.85, UPLSAP 20x Olympus) on a temperature-controlled stage heating system at 37 °C. For each well, phase contrast or bright filed images were taken at 5 randomly selected positions at 5–15 min time intervals over 15 h with an autofocus system using the CellSens Dimensions Software (Olympus). The time-lapse videos were analyzed using Fiji (version 1.49v, U. S. National Institutes of Health, Bethesda, MD, USA). The death time of every single cell undergoing apoptosis, showing membrane blebbing and cell fragmentation, was identified manually (as this resulted in less erroneous results compared to an AI based detection). Frame numbers of each apoptosis event were marked individually and exported to a table. This table was loaded with Matlab (R2018a, The MathWorks, Inc.) and fitted with the Hill equation (see Eq. 1). Here, P_max_ and P_min_ are the maximal and minimal fractions of apoptotic cells, and t_half_ is the time when half of all cells die. n is the Hill coefficient indicating the slope of the curve, corresponding to the apoptosis rate by which cell death manifests in the population. Standard deviations were calculated from measurement replicates.

1 $$P\left( t \right) = {P_{{\mathbf{max}}}} - \frac{{{P_{{\mathbf{max}}}} - {P_{{\mathbf{min}}}}}}{{1 + {{\left( {{\raise0.7ex\hbox{$t$} \!\mathord{\left/{\vphantom {t {{t_{{\mathbf{half}}}}}}}\right.\kern-\nulldelimiterspace}\!\lower0.7ex\hbox{${{t_{{\mathbf{half}}}}}$}}} \right)}^n}}}$$

### DNA origami folding & imaging

DNA origami was folded with 12.5 nM p7249 scaffold (tilibit nanosystems), with 4x excess of staples, 8x excess of staples with anti-handles (purchased from IDT) in TAE (40 mM Tris, 20 mM acetic acid, 1 mM EDTA, pH 8.2), supplemented with 12.5 mM MgCl_2_. The folding mixture was heated to 65 °C and subsequently cooled to 4 °C, over 16 h on a Biometra TRIO (analytik jena GmbH + CoKG). The DNA origami was purified with Amicon centrifugal filters (Merck Millipore, 100 kDa cutoff, cat.no.: UFC5100) and washed five times with TAE supplemented with 5 mM MgCl_2_ at 8000 rcf for 4 min each. ssDNA-IZ-CD95L in 5x excess over each binding site on the DNA origami was added to the DNA origami mixture and incubated overnight at 4 °C. The DNA origami conjugated to ssDNA-IZ-CD95L was again purified with Amicon centrifugal filters (Merck Millipore, 100 kDa cutoff) and washed five times with TAE supplemented with 5 mM MgCl_2_ at 8000 rcf for 4 min each.

### Transmission electron microscopy

Carbon-coated copper grids (Plano GmbH, cat.no.: S162-3) were glow-discharged with oxygen plasma. Then 10 µl of 2 nM C ssDNA-IZ-CD95L on DNA origami were incubated on the grid for 1 min. Excess solution was removed using filter paper. The grid was then stained twice with 10 µl 1.5% uranyl formate solution. TEM imaging was conducted on a Jeol-JEM-1230 operating at an acceleration voltage of 80 kV. TEM micrographs were analyzed with Fiji version 2.14.0 or later.

## Results

To motivate the IZ-CD95L monomer design, we first introduce the native CD95L structure. Figure 1A illustrates the main domains of membrane-bound CD95L monomer. CD95L naturally occurs in a trimeric form, mediated by the THD. A C-terminal domain (amino acids 279–281) is responsible for binding the CD95 receptor. The transmembrane domain enhances the stability of the CD95L trimer, and such stabilization was suggested to play a crucial role in the apoptosis initiation [47]. The PRD influences the expression level and stability of CD95L. The stalk region can be cleaved by various metalloproteinases (MMPs) to form soluble CD95L which turned out to be significantly less efficient in apoptosis initiation than the membrane-bound, stabilized trimer of CD95L [16, 48]. The crystal structure of the CD95L extracellular domain, resolved in previous studies [49], reveals a cone-shaped homotrimer (Fig. 1A right). Within the trimer, each CD95L monomer adopts a jelly-roll fold with two beta-sheets. Trimerization of CD95L further provides an optimal spatial arrangement of the binding sites necessary for high-affinity interaction with CD95. Up to three CD95 can be recruited to CD95L, each binding to the groove between two CD95L monomers. CD95L has three N-linked glycosylation sites at positions Asn184, Asn250, and Asn260 [33, 50], and two native cysteine residues, Cys202 and Cys233, forming an intracellular disulfide bridge that enhances structure stability. Mutagenesis studies [51] have shown that charged and polar amino acids, such as cysteine (Cys) and asparagine (Asn) predominantly mediate CD95-CD95L interactions. In contrast, interactions between monomer subunits involve van der Waals forces and hydrogen bonds located between the beta-sheets.

The plasmid design is shown in Fig. 1B The CD95 ligand extracellular domain (amino acids 137–281) is fused with an IZ domain, as the self-trimerization domain to enhance structure stability and ensure efficient apoptosis induction. This is followed by one extra cysteine for site-specific conjugation (such as biotinylation or DNA conjugation). Additionally, we designed IZ-CD95L either for [1] transient transfection or for [2] stable expression in HEK293T cells. In case of [1], transient transfections, IZ-CD95L was equipped with a His-tag and TEV protease sequence for affinity purification and subsequent cleavage of the N-terminal region. A signal peptide was fused before this sequence at the N-terminus, to allow for secretory expression and to increase the yield. As a cost-efficient reagent for transient transfection in comparison with other commercially available reagents, Polyethylenimine (PEI) was used. PEI was previously shown to be suitable for larger-scale transfection with high efficiency [52, 53]. For the complete transient transfection protocol see IZ-CD95L plasmid design for transient transfections and Large-scale expression of His-IZ-CD95L using cell factory in the Methods section. For comparison, in case [2], a HEK293T cell line stably expressing IZ-CD95L was established, wherefore IZ-CD95L was equipped with a Strep-Tag in combination with a PreScission cleavage site for affinity purification and tag removal. A human VEGF leader sequence was fused before this sequence at the N-terminus, to allow for secretory expression. For the complete protocol see Formation of stable cell line and protein purification in the Methods section. In the following, we focus on case [1], as here several measures were taken to improve the yield (to 350 µg / 1 L culture), while in case of [2] the yield is already high by the use of a stably expression cell line (of 2 mg / 1 L culture).

Cell morphology was checked before transfection and monitored daily afterwards (Fig. 1C upper panel). Following the transfection, cells changed from a single, separated shape to a clustered, cloud-like shape, and the culture medium became cloudy. Cell culture medium was collected 72 h post-transfection. Naturally, also the number of floating cells (dead cells) increased over time. Figure 1C lower panel shows HEK293T cells transfected by PEI/DNA in a 10-layer cell factory in the cell culture hood (left) and cell culture incubator (right). We chose to use the cell factory due to the following advantages: first, it greatly saves time, space, and material in scaling up cell numbers in comparison to the use of, e.g. T175 flasks, since its surface area of 6360 cm^2^, which is equivalent to 36 T175 flasks. It further leads to a lower contamination risk. Second, usage of the cell factory is based on a regular adherent cell culture routine, which does not need additional laboratory equipment. The His-IZ-CD95L expression and secretion was verified by WB using both, the anti-CD95L antibody (Fig. 1D) and the anti-His-tag antibody (Figure S1 lane 1) resulting in a signal band at ~ 35 kDa of the monomeric His-IZ-CD95L.

**Fig. 2:**
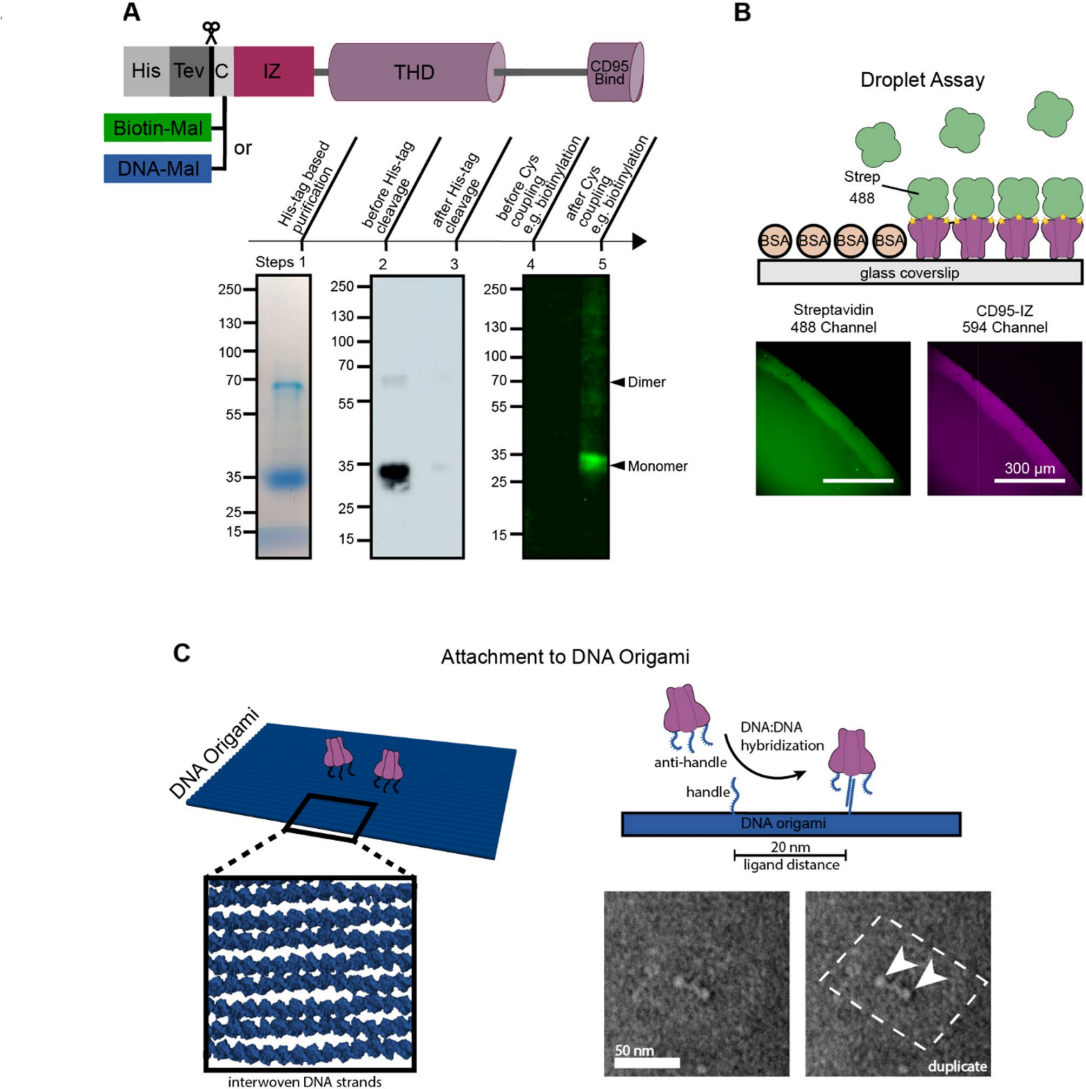
Purification, modification, and structural analysis of IZ-CD95L. **A** Lane 1 Affinity purification of His-IZ-CD95L using anti-His-tag antibody coupled agarose beads checked by 10% SDS-PAGE. Lane 2–3 Western blot analysis using anti-His-tag antibody: 100 ng His-IZ-CD95L before (lane 2) and 100 ng IZ-CD95L after His-tag cleavage (lane 3). Lane 4–5 Western blot analysis of site-specific biotinylation using the cysteine maleimide reaction: 300 ng IZ-CD95L before (lane 4) and after (lane 5) biotinylation were loaded, probed with AF488-streptavidin. **B** Droplet assay to verify biotinylation. Top: a sketch of the assay. Bottom: Fluorescence colocalization images of the 488 nm and the 594 nm channel. Biotin-IZ-CD95L was non-specifically labeled with ATTO594 NHS-ester and incubated with AF488-streptavidin on a BSA passivated glass coverslip. **C** DNA origami functionalized with ssDNA-IZ-CD95L. The DNA handle on the IZ-CD95L hybridizes to a complementary anti-handle on the DNA origami, attaching the ligand site-specifically to the DNA origami. The TEM micrograph on the lower right is duplicated and origami outlines and proteins are indicated for better interpretability

By employing affinity purification using anti-His-tag antibody coupled agarose beads, we obtained an efficient yield of 455$\:\:\mu\:g$ from 1.3 L medium of human His-IZ-CD95L, enabling downstream modifications such as His-tag cleavage, biotinylation, DNA hybridization, or fluorescent labeling. Agarose beads were used in a batch procedure and elution was achieved by an alkaline elution method based on a calculated high theoretical isoelectric point (pI) of His-IZ-CD95L (pI 8.8). Figure 2A lane 1 shows the SDS-PAGE result with a calculated purity of ～86%. The monomeric molecular weight (M.W.) of the secreted His-IZ-CD95L without modification (i.e. with His-tag but without the cleaved signaling peptide) is calculated as 24.1 kDa. On SDS-PAGE the monomer His-IZ-CD95L runs at a molecular weight of 35 kDa due to glycosylation (Fig. 2A lane 1). N-linked glycans can add substantial mass to the protein, typically ranging from 1 to 3 kDa per glycan, and even higher in more complex forms. The observed 11 kDa difference suggests the presence of three to five N-glycans in IZ-CD95L. Furthermore, glycosylation is often heterogeneous, resulting in different glycan structures on individual proteins, which manifests itself as a smear or multiple bands in the SDS-PAGE (Fig. 2A lane 1). Additionally, dimers were observed around 70 kDa, which persisted despite heating and addition of the TCEP reducing agent. These dimers may arise from strong hydrophobic interactions, ionic and hydrogen bonds between CD95L monomers that cannot be disrupted by SDS molecules. The dimers can be observed from previous purification results [26, 29, 30]. Overall, this observation suggests a particularly stable dimeric structure.

After 3 days of secretion expression, the cell pellet was collected and tested by western blot (Fig. 1D). There was a great amount of His-IZ-CD95L identified in the cell lysate. However, to ensure the proper folding and cleavage of signal peptide, the cellular parts of ligands was not used. The biological activity of His-IZ-CD95L before purification was assessed by incubating the collected medium with Hela WT cells, estimating the ligand concentration to be approximately 2000 ng/ml (Figure S3A). The His-IZ-CD95L protein concentration in the expression medium was estimated ～1000 ng/ml, calculated from the band intensity from western blot (Fig. 1D). Since the apoptosis dynamics curve of the unpurified ligand (Figure S3A) showed an apoptosis induction efficiency of 100% at long time scales. The estimated 2000 ng/ml ligand concentration may hence be higher, with the difference arising from protein loss during the preparation steps. Since IZ-CD95L with and without His-tag differs only by a M.W. of 1.8 kDa, no visible size differences were expected and also observed in the SDS-PAGE and western blot used to test for the His-tag cleavage. After His-tag cleavage (Fig. 2A lane 3) a significant reduction in the signal intensity in comparison to the signal before cleavage (Fig. 2A lane 2) is observed, yielding a high calculated cleavage efficiency of 98%.

IZ-CD95L contains two native cysteines that form intracellular disulfide bridges crucial for its structural stability [51]. While the reducing environment of the cytoplasm maintains cysteine residues in their thiol(-SH) form, the additional unpaired cysteine fused at the N-terminus can undergo a non-specific oxidation either during post-secretion or during purification under non-reduced conditions. Therefore, TCEP was used to ensure the accessibility of the thiol group for subsequent maleimide-C5-biotin conjugation. This labeling reaction did not impair the CD95 binding activity or apoptosis induction, as confirmed by subsequent assays (Fig. 4A and C) and is consistent with previous studies [54–56]. Biotinylation was initially validated by western blot using AF488-streptavidin, showing the absence of a band for IZ-CD95L without biotin (Fig. 2A lane 4) and presence of a band after biotinylation (lane 5). Biotinylation was further tested by a droplet assay (Fig. 2B**).** Here, colocalization of AF488-streptavidin (green) and ATTO594-biotin-IZ-CD95L (violet) verified the accessible biotin fused to IZ-CD95L.

Next to biotin functionalization, we demonstrate the specific attachment of ssDNA to the IZ-CD95L, as confirmed by denaturing SDS-PAGE (Figure S4) and by attachment to a DNA origami (Fig. 2C) [57]. A ratiometric analysis of band intensities shows, that approximately 2/5 of the IZ-CD95L monomers exhibit a ssDNA modification. The amount of homotrimers labeled at least once, *P*_*labeled*_, can then be calculated via the binomial distribution as:

$${P_{labeled}} = 1 - {\left( {\frac{3}{5}} \right)^3} = 78.4\% $$

This is the amount of ssDNA-IZ-CD95L homotrimers, which is capable to attach to the DNA origami. The ssDNA-IZ-CD95L homotrimer was then attached to a DNA origami, as depicted in Fig. 2C. Complementary to the ssDNA sequence on the ssDNA-IZ-CD95L, two ssDNA strands were positioned on the DNA origami with a distance of 20 nm. The DNA-IZ-CD95L origami construct was then verified by TEM imaging (Fig. 2C and Figure S5). The probability of ssDNA-IZ-CD95L attachment to the DNA origami was determined by evaluating the TEM micrographs. To avoid inaccuracies that arise with inadvertently discarding badly visible origami, we determined the probability of attachment *p*_*attached*_ by means of stochasticity: the number of DNA origami with one (*N*_*one*_ = 18) and two (*N*_*two*_ = 23) ssDNA-IZ-CD95L attached were counted (Figure S5) and the respective combinatorial combinatorial equations

2 $$\begin{aligned}\frac{{{N_{one}}}}{N} & = 2\left[ {{p_{attached}}\left( {1 - {p_{attached}}} \right)} \right] \\ & = 2{p_{attached}} - 2{p_{attached}}^2 \\ \end{aligned} $$

3 $$\frac{{{N_{two}}}}{N} = {\left( {{p_{attached}}} \right)^2}$$

solved for p_attached_, with *N* = *N*_*one*_*+ N*_*two*_ = 41. The attachment probability was determined to be ***p***_***attached***_ ≅ **71.2%.** N.B.: The calculated probability of attachment might change if the DNA origami without proteins could be reliably identified. Further, if the probability of attachment was corrected by the underlying incorporation rate of the respective ssDNA handle into the origami, which is approximately 85% [57], the probability of attachment for the ssDNA-IZ-CD95L would increase to over 80%.

**Fig. 3:**
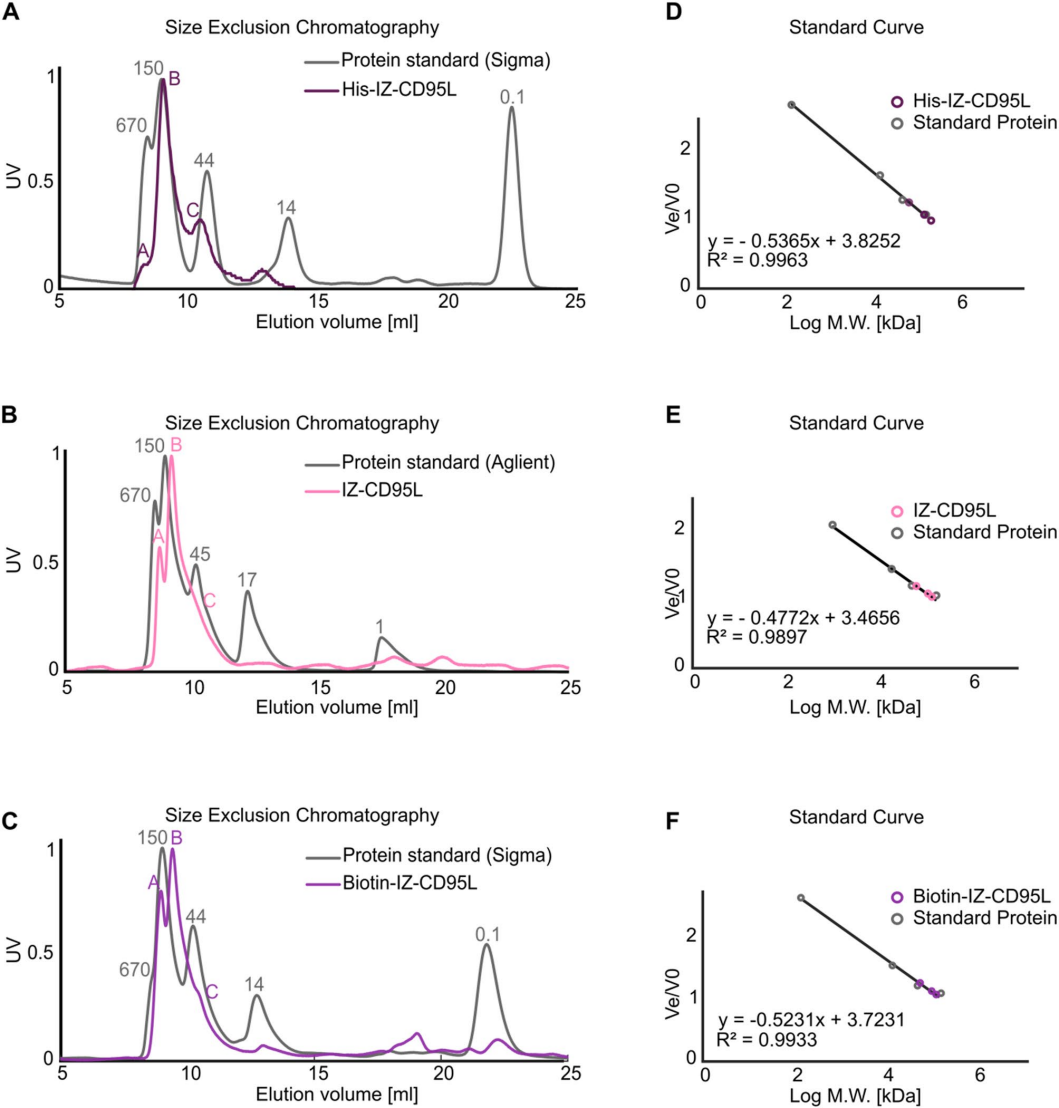
Size exclusion chromatography analysis of X-IZ-CD95L. SEC analysis of His-IZ-CD95L was shown in **A**, IZ-CD95L in **B**, and Biotin-IZ-CD95L in **C**. In all cases, three peaks (peak **A**, **B**, **C**) can be distinguished from X-IZ-CD95L elutions. Two types of protein standard mix were analyzed: protein standard mix 15–600 kDa (Sigma) is shown in **A** and **C**. AdvanceBio SEC Standards (Aglient) is shown in **B**. Standard curves were built in **D-E-F** from **A**-**B**-**C** with X-IZ-CD95 size calculation

Figure 3 presents the size exclusion chromatography (SEC) analysis of His-IZ-CD95L (Fig. 3A), IZ-CD95L (Fig. 3B), and biotin-IZ-CD95L (Fig. 3C), using two different protein standard mixtures and chromatography systems.

Figure 3A shows the separation of a protein standard mix (Sigma; 670 kDa, 150 kDa, 44 kDa, 14 kDa, and 0.1 kDa) on a Superdex 75 column using the Äkta Prime system. Figure 3B depicts the separation of a different protein standard mix (Agilent; 670 kDa, 150 kDa, 45 kDa, 17 kDa, and 1 kDa) on the Enrich 300 column with the NGC Bio-Rad system. Figure 3C presents the protein standard mix from Sigma analyzed on an Enrich 300 column using the NGC Bio-Rad system. The identification of protein standards was validated by SDS-PAGE (Figure S2D-E). Elution volumes (V_e_/V_0_​) of the protein standards are listed in Table 1, with their log molecular weights and V_e_/V_0_​ ratios plotted in Fig. 3D-F. Using these data, the molecular weights of the proteins were calculated, as summarized in Table 2. SEC analysis revealed three distinguishable peaks for X-IZ-CD95L in Fig. 3A and B, and 3C. The identities of these peaks were confirmed by western blotting (Figure S2F). Peak A corresponds to high molecular weight multimers, as evidenced by the non-reducing western blot (Figure S2F, lane 7). This was also observed by cross-linking of His-IZ-CD95L with 4% FA (Figure S1 lane 4), presumably attributed to intermolecular cysteine interaction or a so-called domain-swapping mechanism of isoleucine zipper, as proposed in [58].

Peak B represents the trimeric form of X-IZ-CD95L, while peak C corresponds to the dimeric form. The trimeric form (Peak B) is the dominant species, accounting for 58% in His-IZ-CD95L, 55% in IZ-CD95L, and 48% in biotin-IZ-CD95L from Gaussian fitting results in FigureS2A-C. This distribution is in line with western blot results obtained under non-reducing conditions (Figure S1 lane 3), resolving monomers, dimers, and trimers of His-IZ-CD95L with apparent molecular sizes of approximately 36 kDa, 63 kDa, and 110 kDa, respectively. This is in line with SEC result of IZ-CD95L peak B as trimer (101 kDa) and peak C as dimer (58 kDa) as shown in Table 2. Although flow rate (0.3 ml/min) and running buffer were kept the same, SEC results were different between purification systems and columns (Table 2). The protein purity following SEC purification increased to 97% (Figure S1 lanes 5–6).

**Table 1:** The standard curve is calculated from the elution volumes and molecular weight of the protein standard mix. With V_e_ = elution volume (ml), V_o_ = void volume (ml), V_e_ / V_0_, molecular weight (M.W., kDa)

SEC system and column	Protein name	V_e_	V_e_/V_0_	M.W.	Log M.W.
Protein standard mix 15–600 kDa (Sigma) Superdex 75 10/300 GL (Cytiva) Äkta prime plus (Cytiva)	Thyroglobulin bovine	8.40	1.00	670.00	5.83
	γ-globulins	8.96	1.07	150.00	5.18
	Albumin chicken egg	10.71	1.28	44.30	4.65
	Ribonuclease A	13.86	1.65	13.70	4.14
	pABA (para-aminobenzoic acid)	22.46	2.67	0.14	2.14
AdvanceBio SEC Standards (Agilent) ENrich Sects. 70 10/300 column (Biorad) NGC chromatography system (Biorad)	Thyroglobulin bovine	8.40	1.00	670.00	5.83
	γ-globulins	8.96	1.06	150.00	5.18
	Albumin chicken egg	10.71	1.19	44.30	4.65
	Ribonuclease A	13.86	1.44	13.70	4.14
	pABA (para-aminobenzoic acid)	22.46	2.08	0.14	2.14
Protein standard mix 15–600 kDa (Sigma) ENrich Sects. 70 10/300 column (Biorad) NGC chromatography system (Biorad)	Thyroglobulin bovine	8.40	1.00	670.00	5.83
	γ-globulins	8.96	1.10	150.00	5.18
	Albumin chicken egg	10.71	1.24	44.30	4.65
	Ribonuclease A	13.86	1.55	13.70	4.14
	pABA (para-aminobenzoic acid)	22.46	2.66	0.14	2.14

**Table 2:** X-IZ-CD95L size estimation from the elution volume following the standard curve of the protein standard mix. With V_e_ = elution volume (ml), V_o_ = void volume (ml), V_e_ / V_0_, molecular weight (M.W., kDa)

X-IZ-CD95L	V_e_	V_e_/V_0_	M.W.
His-IZ-CD95L peak A	8.30	0.99	194
His-IZ-CD95L peak B	9.02	1.07	134
His-IZ-CD95L peak C	10.43	1.24	65
IZ-CD95L peak A	8.75	1.02	132
IZ-CD95L peak B	9.22	1.08	101
IZ-CD95L peak C	10.22	1.19	58
Biotin-IZ-CD95L peak A	8.95	1.08	114
Biotin-IZ-CD95L peak B	9.4	1.13	90
Biotin-IZ-CD95L peak C	10.42	1.25	52

**Fig. 4:**
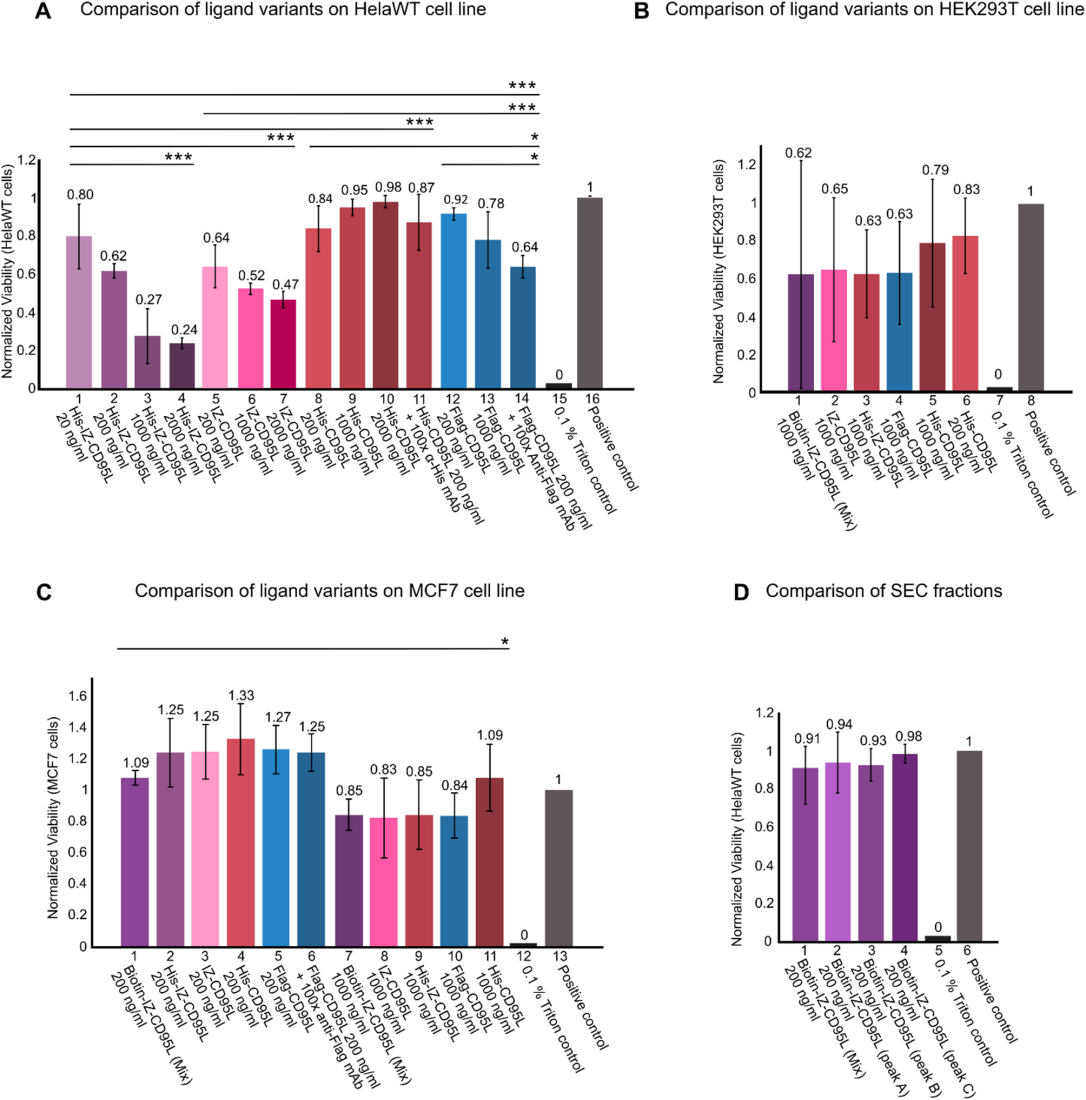
CTB cell viability assay. **A**. Comparison of His-IZ-CD95L, IZ-CD95L, His-CD95L (Prospec) and Flag-CD95L (Enzo) on HelaWT cell line. Lane 1–4: His-IZ-CD95L at concentration variations 20 ng/ml, 200 ng/ml, 1000 ng/ml, 2000 ng/ml. Lane 5–7: IZ-CD95L at 200 ng/ml, 1000 ng/ml, 2000 ng/ml. Lane 8–11: His-CD95L (Prospec) at 200 ng/ml, 1000 ng/ml, 2000 ng/ml, and His-CD95L cross-linked with 100x α-His mAb. **B**. Comparison of ligand variants on HEK293T cell line. **C**. Comparison of ligand variants on MCF7 cell line. **D**. Comparison of SEC fractions. Biotin-IZ-CD95L fraction peaks A-C collected from SEC analysis (Fig. 3C) and tested at 200 ng/ml. In all conditions: 0.1% Triton X-100 was used as negative control and no treatment as positive controls. Error bars were calculated from three biological replicates. Determination of significance was via one way ANOVA (analysis of variance) test, with * *p* < 0.05, ** *p* < 0.01, *** *p* < 0.001. Detailed results are shown in table S1

Figure 4 presents the CTB assay results for CD95L variants at varying concentrations in HeLa WT, HEK293T and MCF7 cell lines. Figure 4A lane 1–4 illustrates the increasing cytotoxicity of purified His-IZ-CD95L with concentration-dependency at 20 ng/ml − 2000 ng/ml, which is not significant in lane 5–7 of IZ-CD95L. His-CD95L was tested as control shown in lane 8–11, with no concentration dependency observed even after anti-His mAb cross-linking. His-IZ-CD95L exhibit better pro-apoptotic effects in Hela WT cell line at high concentrations (1000 ng/ml, 2000 ng/ml), suggesting a positive role of fusion His-tag additionally on the N-terminus of IZ-CD95L. However, fusion with His-tag alone does not show superior effects (Fig. 4A lane 8–11). Flag-CD95L showed enhanced cytotoxicity after cross-linking with 100x anti-Flag mAb (lane 14). Figure 4B and C show ligand variants effect on HEK293T and MCF7 cell lines. At 200 ng/ml of ligand variants, there is no cytotoxic effect observed in the MCF7 cell line. However, at 1000 ng/ml decreased viability is observed for the MCF7 cell line with no significant difference between His-IZ-CD95L and IZ-CD95L. In Fig. 4D Lanes 1–4 compare the cytotoxic effects of different fractions collected from the SEC results in Fig. 3C. No significant differences were observed among peak A (multimer), B (trimer), and C (dimer). Besides, no significant impact on cytotoxicity was observed from 0, 10, 20, and 50 freeze thaw cycles.

**Fig. 5:**
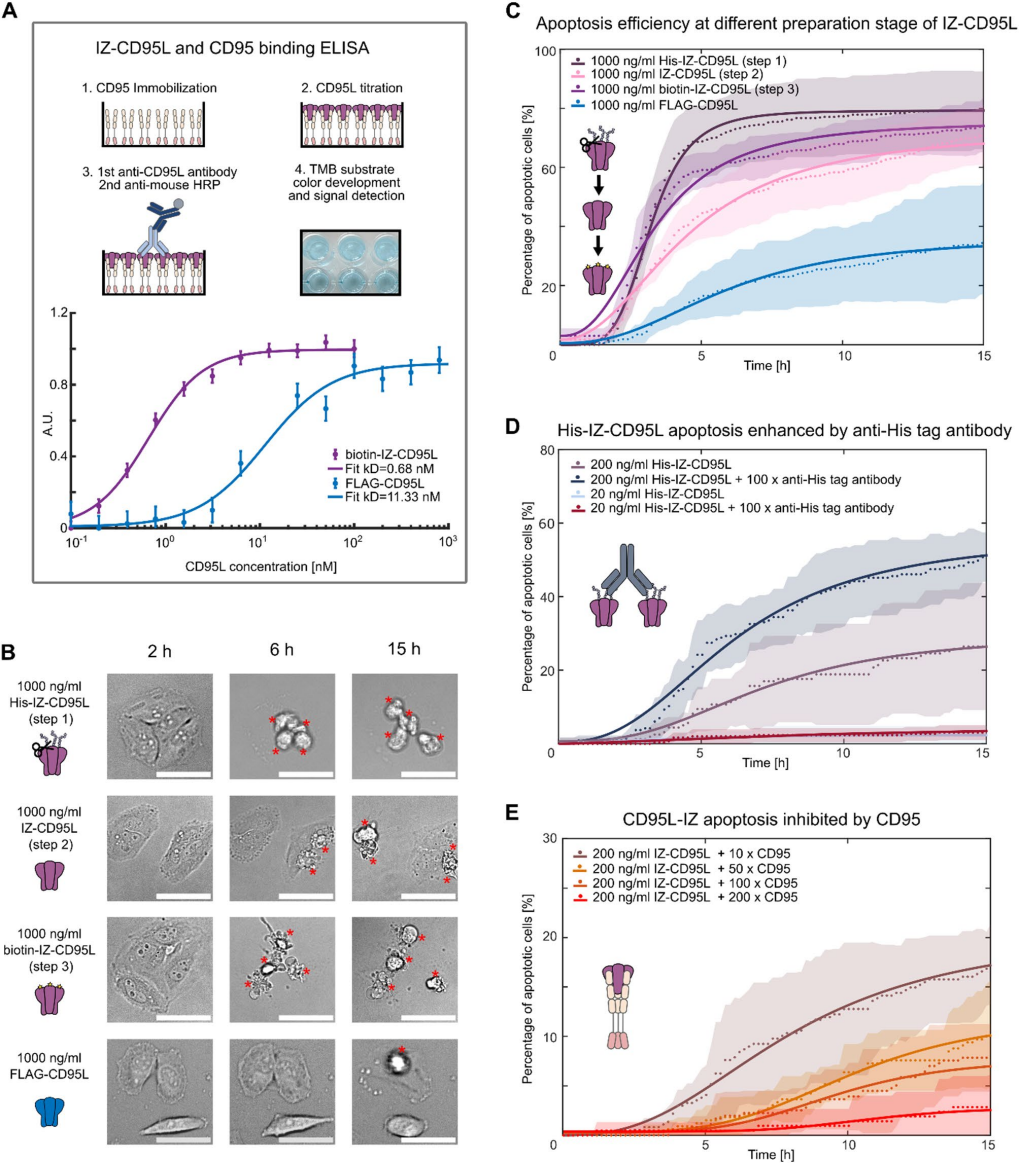
ELISA and apoptosis dynamics assay. **A** ELISA analysis of biotin-IZ-CD95L and FLAG-CD95L binding to CD95. **B** Comparison of representative bright field images showing morphological changes of cells treated with X-IZ-CD95L or FLAG-CD95L after 2, 6, and 15 h. Red asterisks indicate the position of apoptotic cell. Scale bars are 50 μm. **C** Apoptosis dynamics analysis of IZ-CD95L apoptosis induction at different stages: post-purification, post-His-tag cleavage, post-biotinylation, and comparison with FLAG-CD95L. **D** The efficiency of His-IZ-CD95L apoptosis induction is increased by anti-His-tag antibody crosslinking. **E** The presence of CD95-His (in 10x, 50x, 100x, and 200x molar excess in comparison to biotin-IZ-CD95L as competitive binders to the natural CD95 on the cell membrane, successively inhibits biotin-IZ-CD95L apoptosis induction. In panels **C**,** D**, and **E** data points are marked with dots. Hill equation fit curves are represented by solid lines, and standard deviations are indicated with shaded regions

To verify the biological activity of biotin-IZ-CD95L and FLAG-CD95L and its binding to CD95-His, we first performed enzyme-linked immunosorbent assays (ELISA) or native PAGE analysis, as shown in Fig. 5A and Figure S6. From the ELISA binding assay, the dissociation constant (kD) can be derived, with 0.68 nM of biotin-IZ-CD95L and 11.33 nM of FLAG-CD95L (ENZO). This is consistent with previously reported kD value from SPR measurements [59]. Thus, a more than 10-fold higher binding affinity of biotin-IZ-CD95L in comparison to FLAG-CD95L binding to CD95 is obtained, when applied under identical conditions. In Figure S6A, the binding of CD95-His with biotin-IZ-CD95L is demonstrated by a band shift in lane 1, indicated by the red asterisk. In Figure S6B lane 1, CD95-His shows the expected monomer signal band at an apparent molecular weight of 44 kDa as well as a broad signal band around 240 kDa, indicating a multimeric form of CD95. This multimeric form occurs at high concentrations under in vitro native conditions, e.g. a SDS-stable CD95 complex around 200 kDa was also reported in reference [60]. Lane 2–3 show the effect of binding FLAG-CD95L or biotin-IZ-CD95L to CD95-His, which results in slight shifts of the signal bands to higher molecular weights indicated by asterisks.

In Fig. 5C the potency of X-IZ-CD95L to trigger the cell apoptosis pathway was evaluated at different preparation stages: freshly purified His-IZ-CD95L with His-tag, after His-tag cleavage, after biotinylation, and in comparison, to the FLAG-CD95L. Nonspecific cell killing was excluded by control experiments. For example, L-15 complete medium without ligand secretion from HEK293T cells after three days of culture was collected and incubated with Hela WT cells, to rule out the killing effect from other toxic substances expressed by HEK293T cells. As expected, no cell death was observed. Additionally, the secreted ligand in L-15 medium after three days was incubated on Hela CD95^KO^ cell line as a control to exclude non-specific cell killing by other membrane receptor proteins, and no apoptosis was observed in this experiment (Figure S3A).

All parameters, obtained from the apoptosis dynamics analysis are shown in Table 3. In general, X-IZ-CD95L demonstrated around twice the efficiency in initiating apoptosis compared to FLAG-CD95L (Fig. 5C; Table 3) at the same concentration. This can be explained by the higher binding affinity of IZ-CD95L with CD95 (Fig. 5A). As shown in Fig. 5C, a slight decrease in the apoptosis rate n was observed after His-tag cleavage, from *n* = 4.7 to *n* = 2.2. Following biotinylation, the apoptosis rate increased slightly to *n* = 2.5 (Table 3). Similarly, the maximum percentage of apoptotic cells decreased from 79 to 73% after His-tag cleavage and rose again to 76% after biotinylation and the apoptosis half-time increased from 3.2 h to 4.5 h and decreased again to 3.3 h (Table 3). This data shows, that the integrity of CD95L remains intact and that no significant difference among preparation steps exist.

We were then interested to see, if further crosslinking of His-IZ-CD95L by anti-His-tag antibody would increase the maximum apoptosis percentage. As shown in Fig. 5D, while a low concentration of His-IZ-CD95L (20 ng/ml) did not show enhanced apoptosis by antibody crosslinking, a roughly twofold increase in the maximum apoptosis percentage from 29.8 to 56.7% was observed at 200 ng/ml His-IZ-CD95L after crosslinking (Table 3), which has been observed before [21]. This enhancement could be due to several factors: (i) antibody crosslinking may stabilize the ligand and promote a favorable conformation or orientation towards the CD95 receptors. It also (ii) increases the local concentration of IZ-CD95L. Similar effects were previously shown to contribute to a more efficient signal initiation [61, 62]. Finally, we analyzed the apoptosis initiation of IZ-CD95L inhibited by CD95, when IZ-CD95L was pre-incubated with CD95 at excess concentrations (10x, 50x, 100x, 200x) before measuring apoptosis. Here, the maximum percentage of apoptotic cells was successively reduced from 21% to 3% at a 10x and 200x excess of CD95, respectively (see Fig. 5E; Table 3). The reduced efficacy is caused by the sequestration of biotin-IZ-CD95L by excess CD95 and verifies, that IZ-CD95L couples successively more CD95. Here, the added CD95-His acts as a decoy receptor and together with biotin-IZ-CD95L forms inactive complexes that cannot trigger apoptosis anymore.

**Table 3:** Apoptosis rate (n), percentage of maximum apoptotic cells (Max), and half lifetime (T-half) were obtained from fitting with the hill equation

Protein and concentration	*n*	Max (%)	T-half (h)
His-IZ-CD95L 1000 ng/ml	4.7	79.3	3.2
IZ-CD95L 1000 ng/ml	2.2	72.9	4.5
biotin-IZ-CD95L 1000 ng/ml	2.5	75.7	3.3
FLAG-CD95L 1000 ng/ml	2.5	40.6	5.9
His-IZ-CD95L 200 ng/ml	2.8	29.8	7.2
His-IZ-CD95L 200 ng/ml + 100 x anti-His-tag antibody	2.5	56.7	6.2
His-IZ-CD95L 20 ng/ml	1.3	7.7	17.8
His-IZ-CD95L 20 ng/ml + 100 x anti-His-tag antibody	2.2	2.5	3.9
IZ-CD95L 200 ng/ml + 10 x CD95	2.6	20.7	8
IZ-CD95L 200 ng/ml + 50 x CD95	4.1	12.1	10.2
IZ-CD95L 200 ng/ml + 100 x CD95	4.5	7.8	9.5
IZ-CD95L 200 ng/ml + 200 x CD95	5.9	2.9	10.7

## Discussion

In this study, we investigated how bioengineering of CD95L can affect the molecular properties as well as its efficiency to trigger the apoptosis signaling pathway. We chose to fuse an IZ domain to the N-terminus of CD95L in order to stabilize the structure and trimeric state of CD95L. This choice was based on the frequently reported observation that sCD95L is weakly apoptotic and outcompeted by mCD95L to induce cell death and the hypothesis, that mCD95L exhibits a higher structural stabilization and/or orientation of the ligand on the cell membrane. In addition, the oligomeric state appears to play an important role: for example, trimerization of CD95L as confirmed by e.g. gel filtration [14] and cross-linking [15] has been reported in case of competent apoptosis signal initiation. Crosslinking and other structural modifications of biologically functional sCD95L showed to enhance the apoptosis signal initiation. Examples of such modifications are the fusion of a Fc domain [21] resulting in a significant enhancement, leading to ~ 87% apoptosis at a concentration maximum in the Jurkat cell line, compared to 26% for the couple sCD95L form. Further, the fusion of CD95L with small domains such as tenascin-C (TNC) [38] and T4 fibritin (foldon) [62], showed to promote self-trimerization. Another approach includes CD95L fusion with a Flag-tag, which is then crosslinked by anti-Flag-tag antibody [63, 64]. Here, a significant enhancement in the apoptosis initiation efficiency reaching ~67% was observed. was The latter was also implemented in our study for direct comparison with our IZ-CD95L results (see CTB assay in Fig. 4B or Fig. 5). Finally, an early study suggested that two CD95L trimers need to form a dimer to induce apoptosis signaling efficiently [21]. While all studies support that CD95L oligomerization is a crucial factor to induce apoptosis signaling, a quantification of the type and fractions of the appearing oligomers is missing. In our work we show explicitly, how fusion via the IZ motif to CD95L alters the molecular characteristics, such as its oligomeric state (the fraction of trimers, dimers, monomers by size exclusion chromatography analysis in Fig. 3A-C) and binding affinity. We further systematically probe, that not only the higher stability of the trimer due to the IZ motif leads to a signaling enhancement. We also show that this is further amplified (to a twofold increase in the maximum apoptosis percentage) when His-IZ-CD95L is crosslinked via antibodies (Fig. 5C-D). Indeed, the fusion of an isoleucine zipper (IZ) domain to a TNF ligand has been used before to increase the cytotoxicity in case of soluble CD95L [39, 44]. Yet, here no biochemical analysis of the molecule was performed and only in one case IZ-CD95L was purified. IZ fusion was also used in case of CD40L [65, 66], TNFR proteins [67, 68], or TRAIL [69]. Intriguingly, engineered IZ-TRAIL has demonstrated high tumor-specific activity both in vitro and in vivo [70]. In case of IZ-TRAIL a ~80% cytotoxicity of Jurkat cells compared to 10% in gas of FLAG-TRAIL was found. When an anti-FLAG tag antibody was added to FLAG-TRAIL, the cytotoxicity levels could be increased to the level of IZ-TRAIL. Other crosslinking approaches in case of TRAIL include modifications with leucine zipper, and TNC domains [38], as well as anti-FLAGmAb cross-linked FLAG-TNC-TRAIL or stalk-TRAIL. In summary, all studies suggest that spatial fixation of the N-terminus to stabilize the trimeric CD95L configuration is necessary, to amplify the apoptosis signal initiation. Our study further shows that additional crosslinking to create higher-order oligomers further enhances the efficiency of the apoptosis initiation. We infer that the described CD95L modifications result in tighter binding of CD95 as well as a local concentration enhancement of the receptor. This in turn could increase the amount of recruited adaptor molecules and procaspases 8, facilitating the autocatalytic activation of the latter [71]. Finally, as previously suggested, larger amounts of active caspase 8 can result in the immediate activation of effector caspases and a mitochondria-independent apoptosis pathway [3, 72].

Next to stabilizing the trimeric configuration also the orientation of presenting CD95L to the receptor was shown to affect the signal initiation. For example, in previous studies by us and others, CD95L was either anchored to DNA origami in a hexagon geometry and different types of orientations of the anchor were tested [61], or it was coupled on supported lipid bilayers and the concentration of CD95L was varied systematically [61, 62]. In both cases, the orientation and local concentration of CD95L was shown to affect the efficiency of signal initiation. In order to enable further studies on such molecular coupling effects as well as to develop further bionanotechnological or biomedical assays [73], we here demonstrated the successful functionalization of IZ-CD95L with biotin or ssDNA. To this end, we chose to equip IZ-CD95L with a cysteine residue for site-specific chemical modification using maleimide-cysteine reactions. These are considered the most specific labeling reaction due to the high selectivity of the thiol group (-SH). For the biotin modification we conjugated a maleimide functionalized biotin to the cysteine residue, whereas for DNA origami coupling a maleimide functionalized ssDNA strand was used. Via complementary DNA-DNA hybridization, the resulting ssDNA-IZ-CD95L conjugate was then attached to the complementary ssDNA on a rectangular DNA origami sheet. The determined probability of attachment for the ssDNA-IZ-CD95L to the handles was ~ 70%.

The laboratory-based purification and modification protocol presented here for CD95L enables to obtain the protein in ~ mg amounts. This amount is crucial as it allows for affinity-based purification, to perform analyses of the molecular modifications, and to study their effects on the signaling pathway initiation. Using commercially available ligands, such as Flag-CD95L or His-CD95L a characterization of their oligomeric state and functionalization efficiency is hardly possible due to their high cost. We directly compare protein purification from transiently transfected cells versus purification from a stable cell line and provide recommendations how the yield in both cases can be increased. If large protein amounts are required on a regular basis, the establishment of the stable cell line is recommended, as no large amounts of DNA are required and since the yield of the purified protein exceeds the transient transfection yield by a factor ~ 7.

## Conclusions

In this work, we present a recombinant CD95L exhibiting an IZ motif at the N-terminus, which stabilizes the trimerized CD95L. We discuss that this stabilization is necessary to amplify the apoptosis signal initiation and provide corresponding data exposing cancer cells to IZ-CD95L in solution in comparison to the unmodified CD95L. Additional crosslinking of IZ-CD95L with antibodies to create higher-order oligomers further enhances the efficiency of apoptosis initiation. A fast, cheap, and efficient production of this IZ-CD95L *via* the HEK293T secretory expression system is presented, including IZ-CD95L affinity purification. A comprehensive IZ-CD95L characterization confirms the primarily trimeric state, a sub-nM binding affinity, and the complex formation with CD95. A cysteine amino acid fused behind the IZ further served as a versatile coupling site for CD95L functionalization with biotin or ssDNA. Thus, IZ-CD95L with its superior biological activity to trigger the apoptosis signaling pathway, is highly suitable for the development of further bionanotechnological or biomedical assays.

## Electronic supplementary material

Below is the link to the electronic supplementary material.

Supplementary Material 1

Supplementary Material 2

Supplementary Material 3

Supplementary Material 4

Supplementary Material 5

Supplementary Material 6

Supplementary Material 7

Supplementary Material 8

Supplementary Material 9

Supplementary Material 10

Supplementary Material 11

Supplementary Material 12

Supplementary Material 13

Supplementary Material 14

## Data Availability

All data of this study are shown in the main text or the supplementary information. Plasmids are subject to the Uniform Biological Material Transfer Agreement. Requests for the plasmids should be submitted to Cornelia Monzel.

## Abbreviations

CD95: Cluster of Differentiation 95
TNF: Tumor-necrosis factor
IZ: Isoleucine zipper
TNFSF: Tumor necrosis factor super family
SFK: Src-family kinase
mCD95L: Membrane bound CD95 ligand
sCD95L: Soluble CD95 ligand
MMP: Metalloprotease
TM: Transmembrane
THD: TNF homology domain
E.coli: Escherichia coli
COS: Dictyostelium discoideum
2 x YT: 2 x yeast extract tryptone
LB: Lysogeny broth
SDS-PAGE: Sodium dodecyl-sulfate polyacrylamide gel electrophoresis
HEK293: Human embryonic kidney 293
DMEM: Dulbecco’s modified eagle medium
FBS: Fetal bovine serum
P/S: Penicillin/streptomycin
TBS: Tris-buffered saline
TCEP: Tris(2-carboxyethyl)phosphine
PBS: Phosphate-buffered saline
pABA: p-aminobenzoic acid
TEV: Tobacco etch virus
DTT: Dithiothreitol
CD95 ECD: CD95 extracellular domain
BS(PEG)5: PEGylated bis(sulfosuccinimidyl)suberate
PVDF: Polyvinylidene difluoride
BSA: Bovine serum albumin
HRP: Horseradish peroxidase
ECL: Enhanced chemiluminescence
CN PAGE: Clear native polyacrylamide gel electrophoresis
APS: Ammonium persulfate
TEMED: Tetramethylethylenediamine
TE: Tris-EDTA
Sf9/Sf21: Spodoptera frugiperda 9/21
CHO: Chinese hamster ovary
IL2pept: Human interleukin 2
PEI: Polyethylenimine
Ni-NTA: Nickel-nitrilotriacetic acid
TNC: Tenascin-C
Cys: Cysteine
Asn: Asparagine
Ser: Serine
SEC: Size exclusion chromatography
FA: Formaldehyde
TEM: Transmission emission microscopy
pI: Isoelectric point
ELISA: Enzyme-linked immunosorbent assay
TMB: 3,3’,5,5’ - tetramethylbenzidine
CRISPR: Clustered regularly interspaced short palindromic repeats
TRAIL: TNF related apoptosis inducing ligand
