# Supplementary figures and images for: High yield purification of an isoleucine zipper-modified CD95 ligand for efficient cell apoptosis initiation and with biotin or DNA-oligomer binding domain to probe ligand functionalization effects
Source: BMC Biotechnol. 2025 Jul 1;25:64. doi: 10.1186/s12896-025-00986-2 (PMC12219679; doi:10.1186/s12896-025-00986-2)

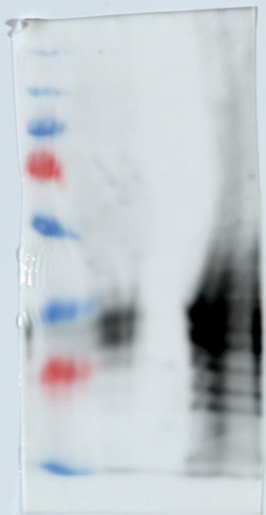

Supplement: Supplementary file 1 — Supplementary Material 1 [file 12896_2025_986_MOESM1_ESM.pdf]

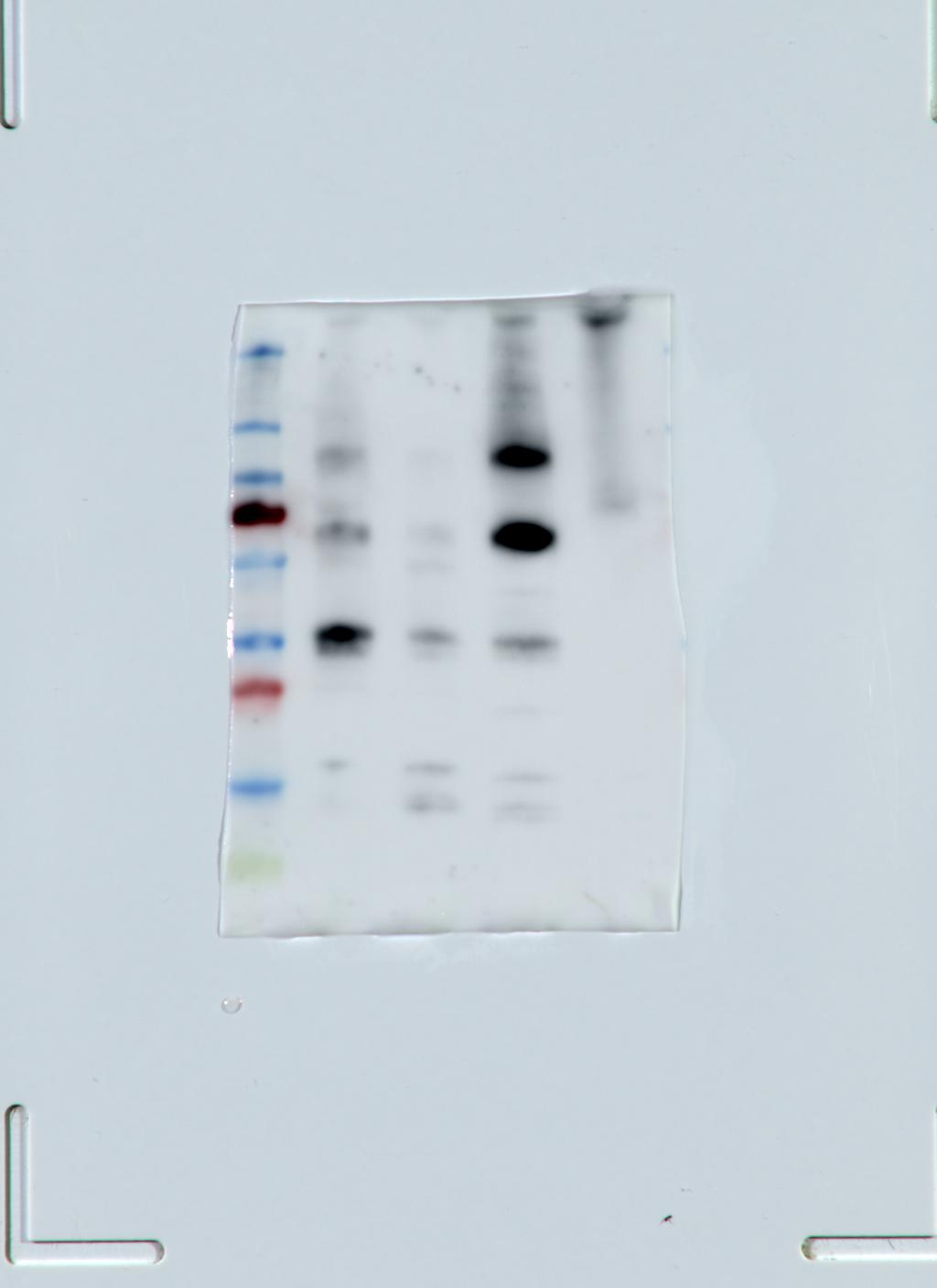

Supplement: Supplementary file 2 — Supplementary Material 2 [file 12896_2025_986_MOESM2_ESM.jpg]

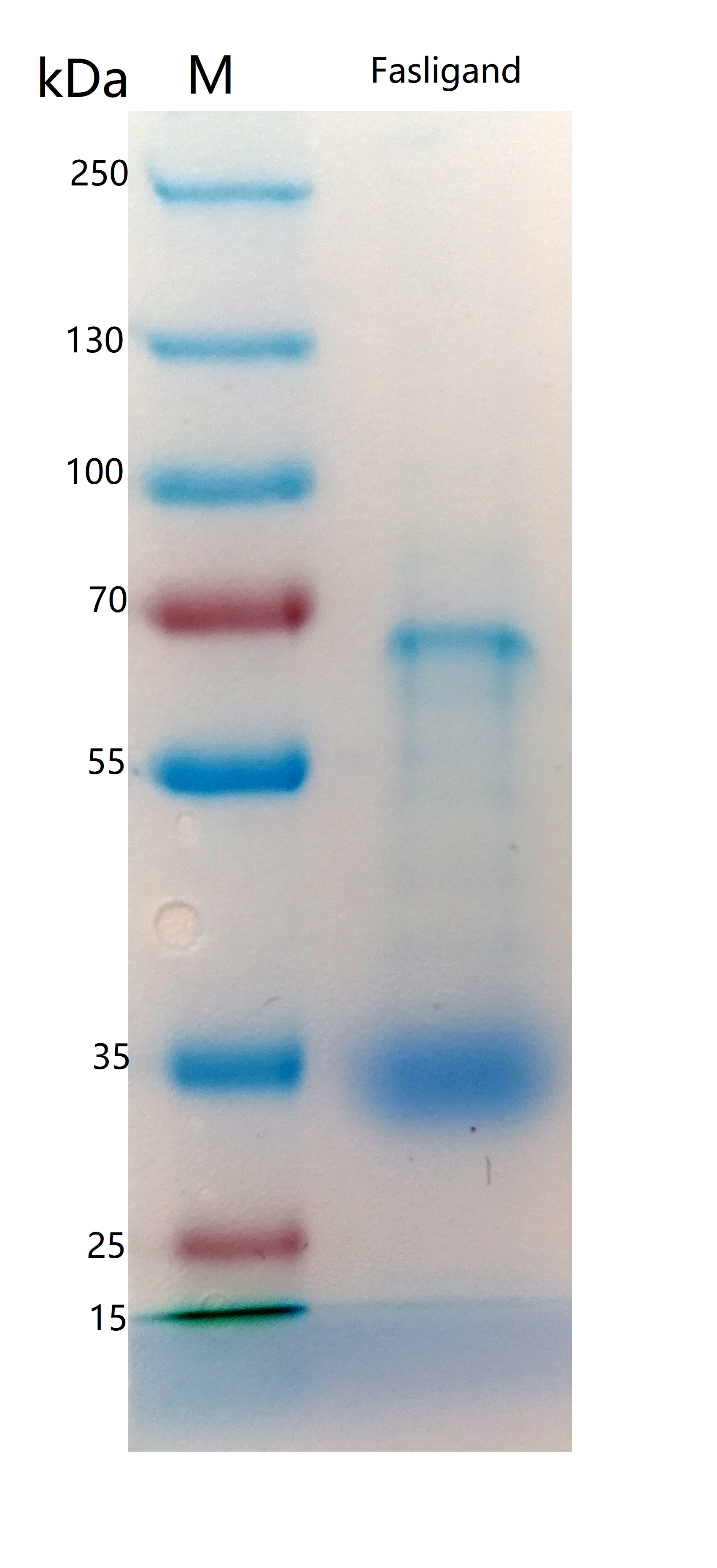

Supplement: Supplementary file 3 — Supplementary Material 3 [file 12896_2025_986_MOESM3_ESM.jpg]

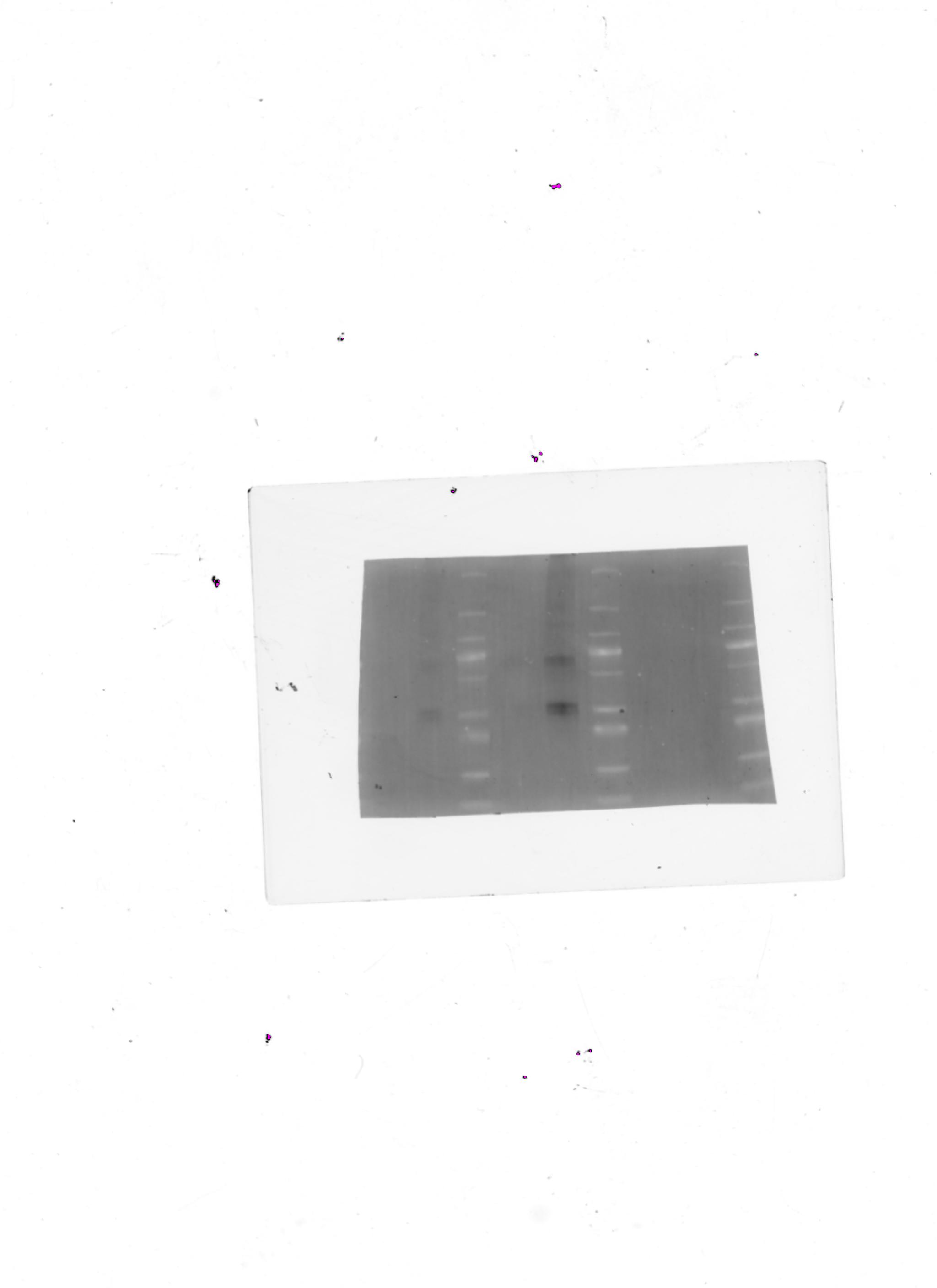

Supplement: Supplementary file 4 — Supplementary Material 4 [file 12896_2025_986_MOESM4_ESM.jpg]

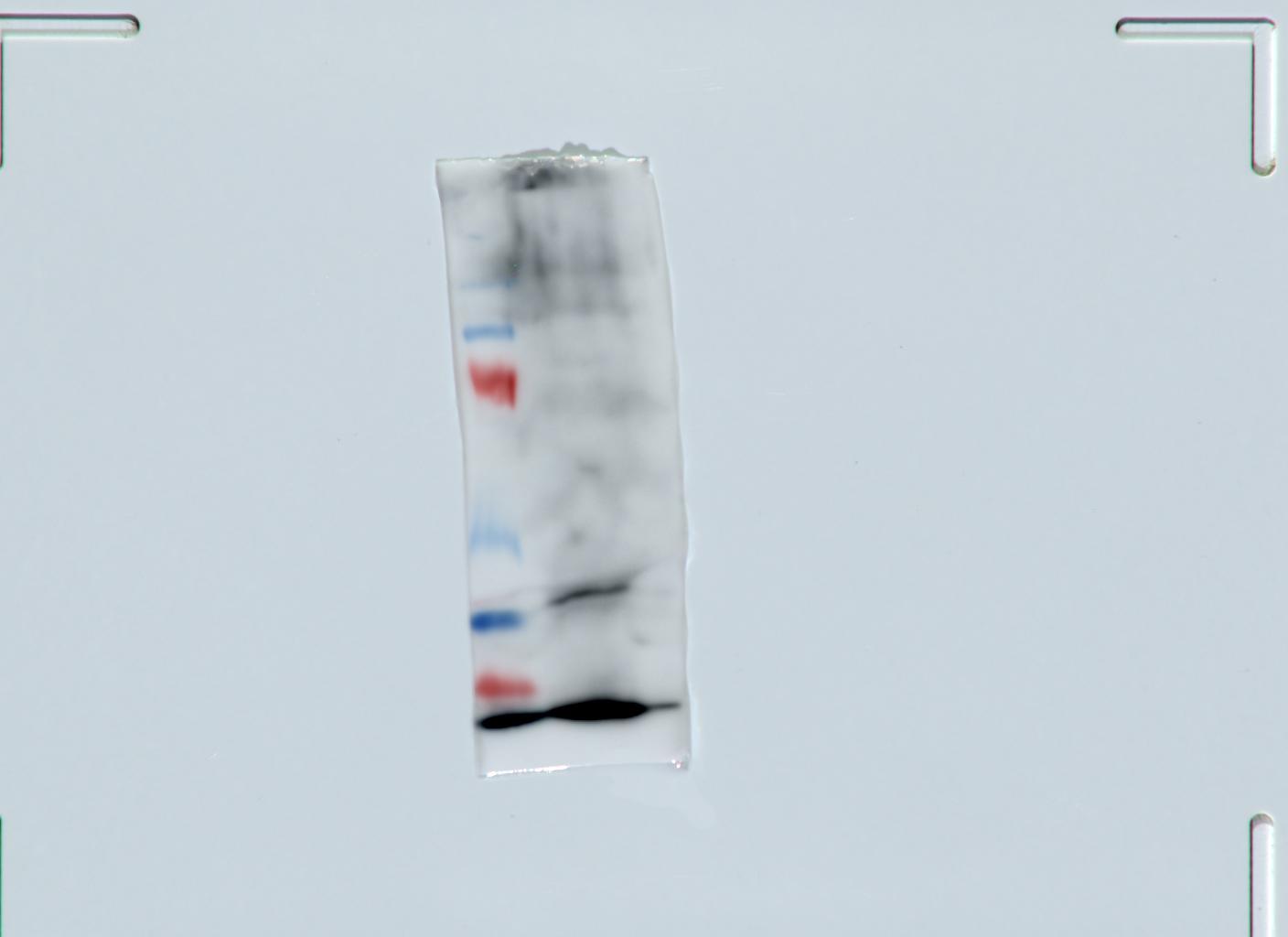

Supplement: Supplementary file 5 — Supplementary Material 5 [file 12896_2025_986_MOESM5_ESM.jpg]

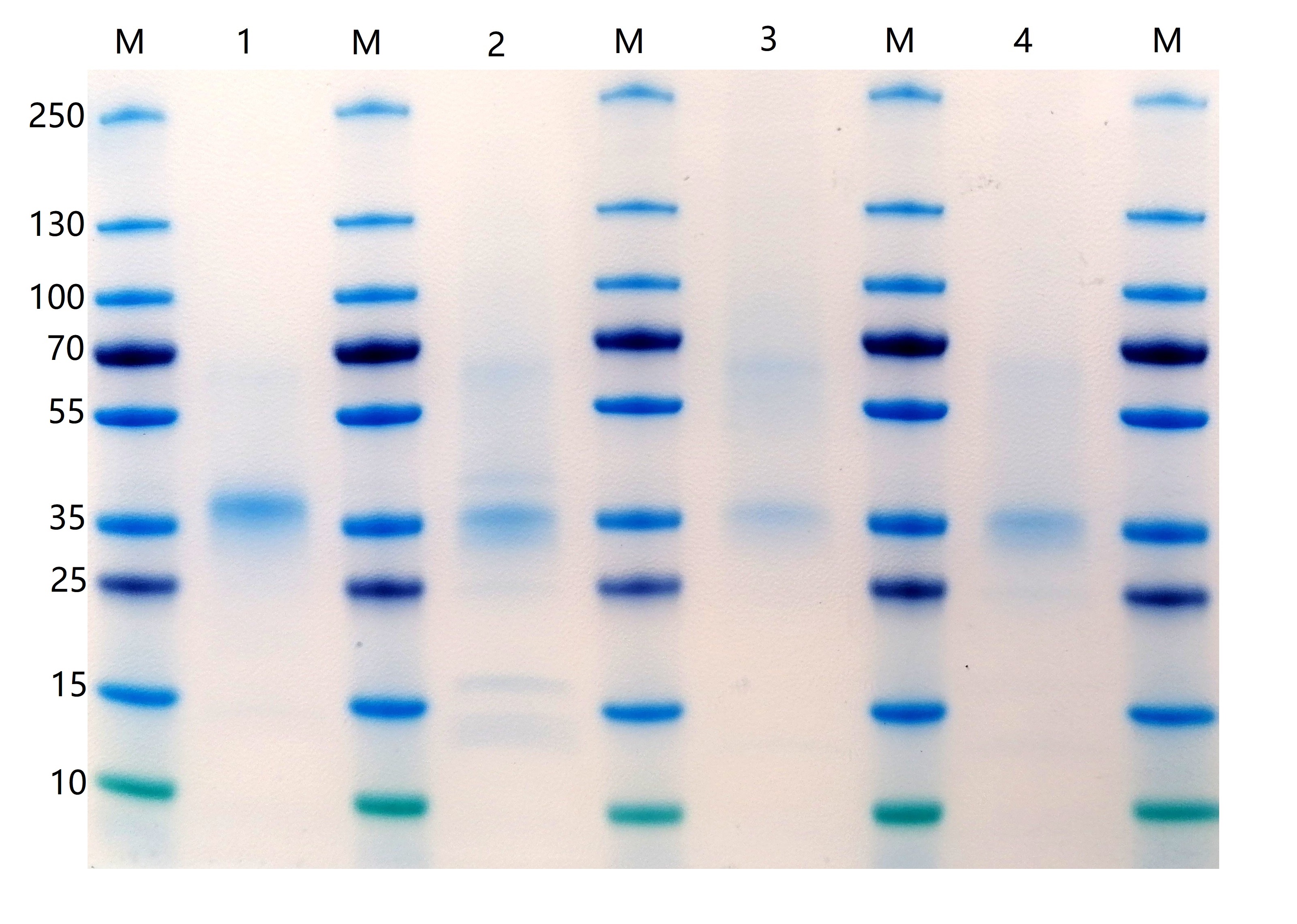

Supplement: Supplementary file 6 — Supplementary Material 6 [file 12896_2025_986_MOESM6_ESM.jpg]

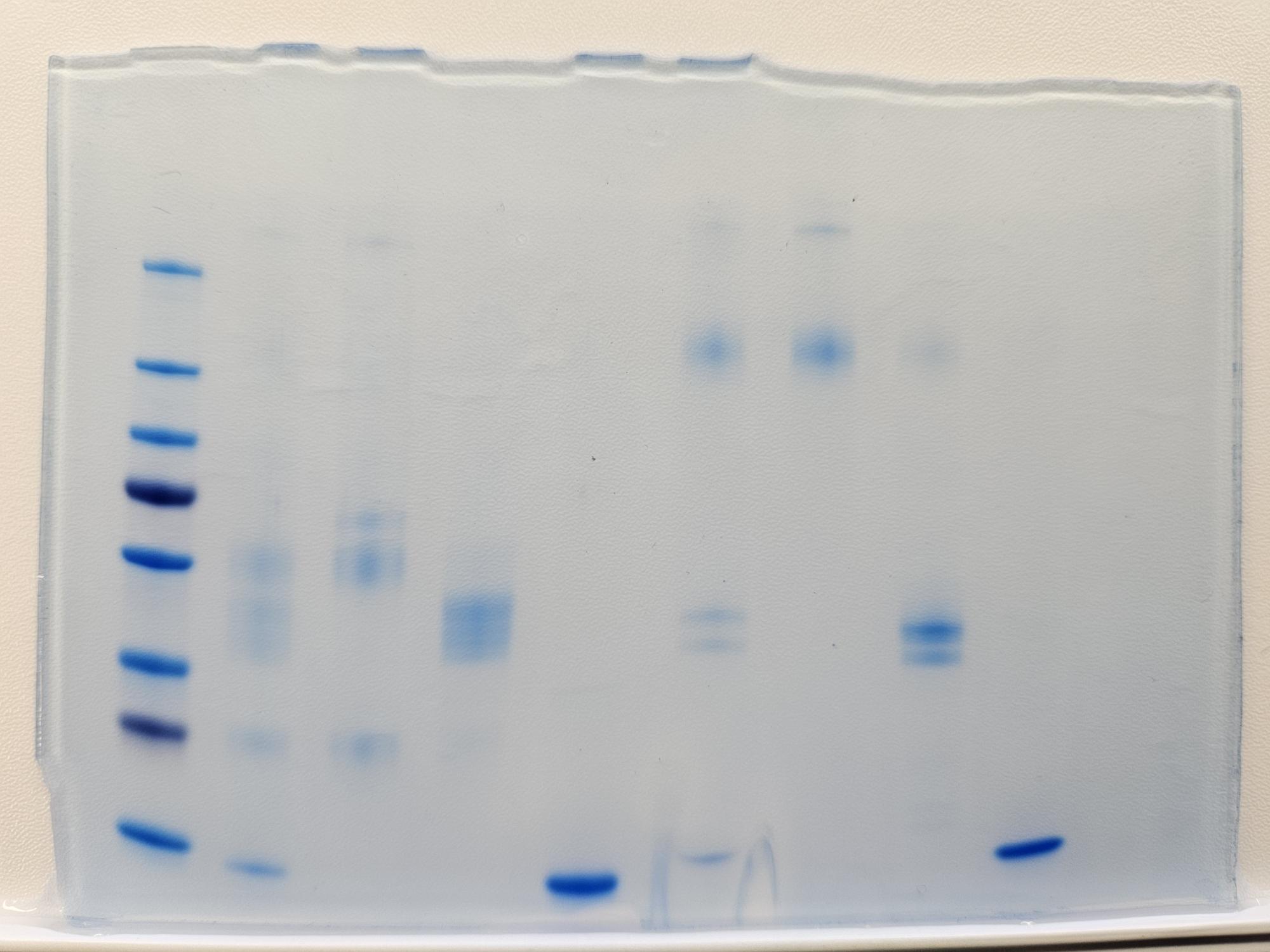

Supplement: Supplementary file 7 — Supplementary Material 7 [file 12896_2025_986_MOESM7_ESM.jpeg]

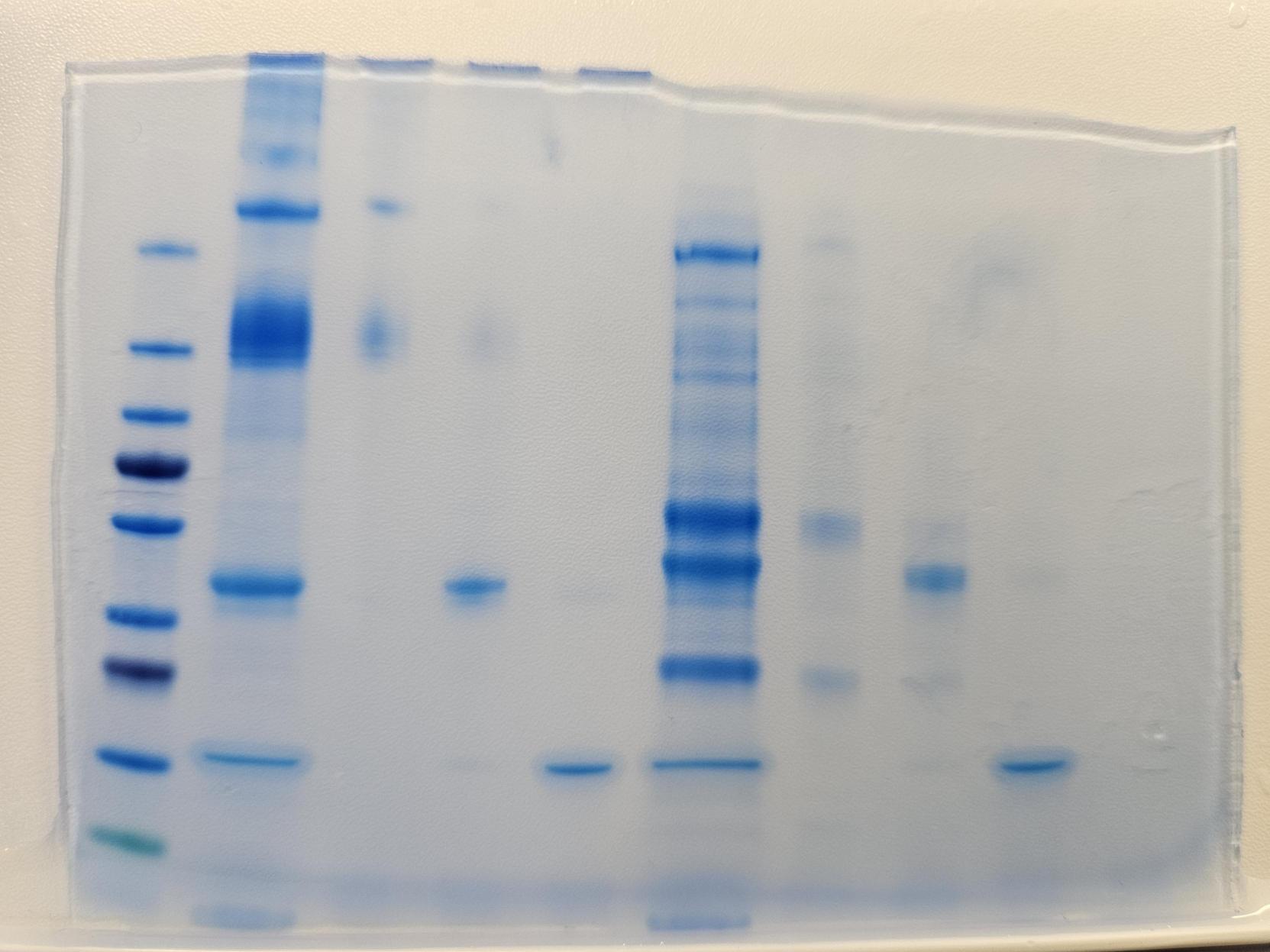

Supplement: Supplementary file 8 — Supplementary Material 8 [file 12896_2025_986_MOESM8_ESM.jpeg]

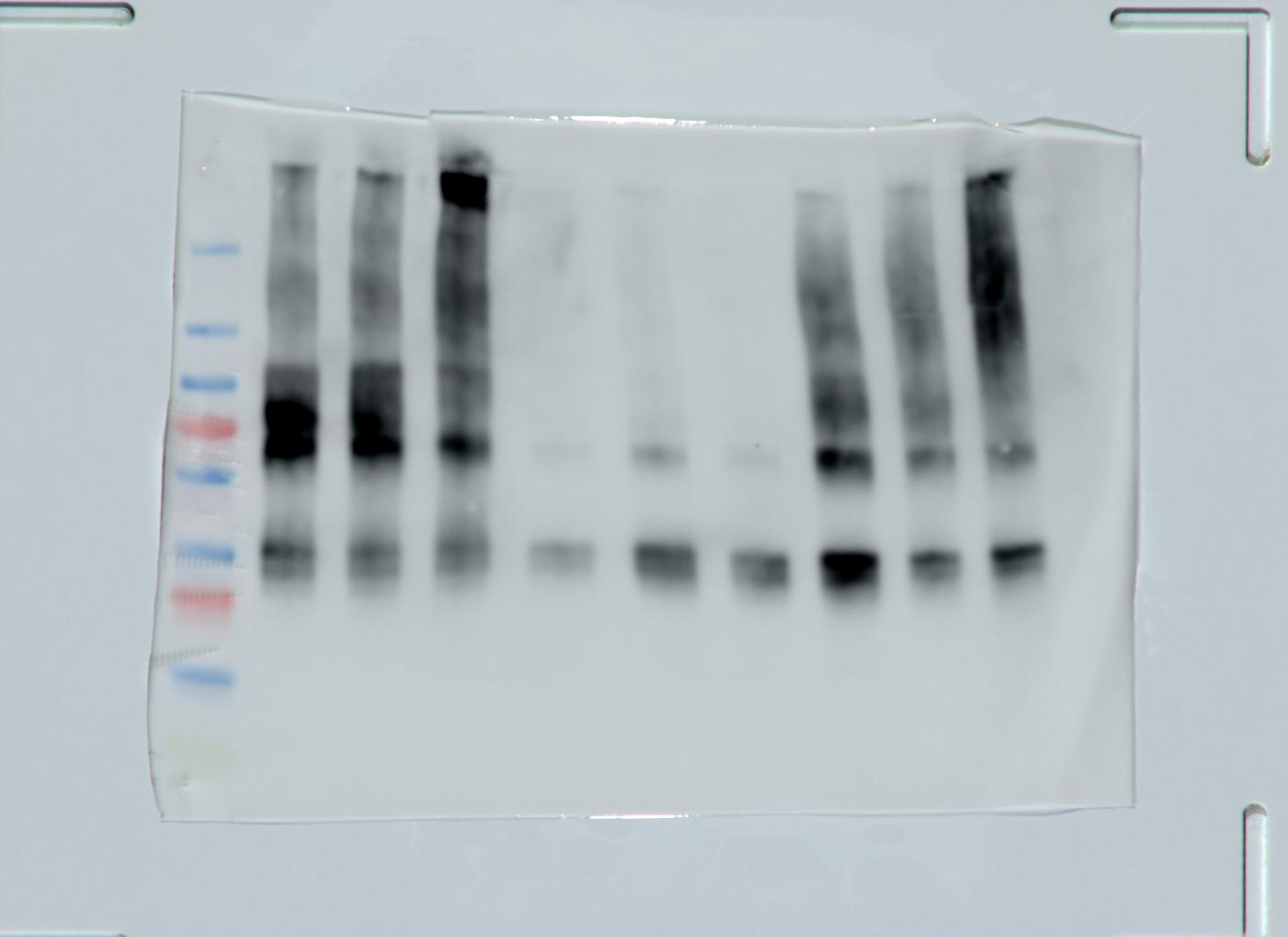

Supplement: Supplementary file 9 — Supplementary Material 9 [file 12896_2025_986_MOESM9_ESM.jpg]

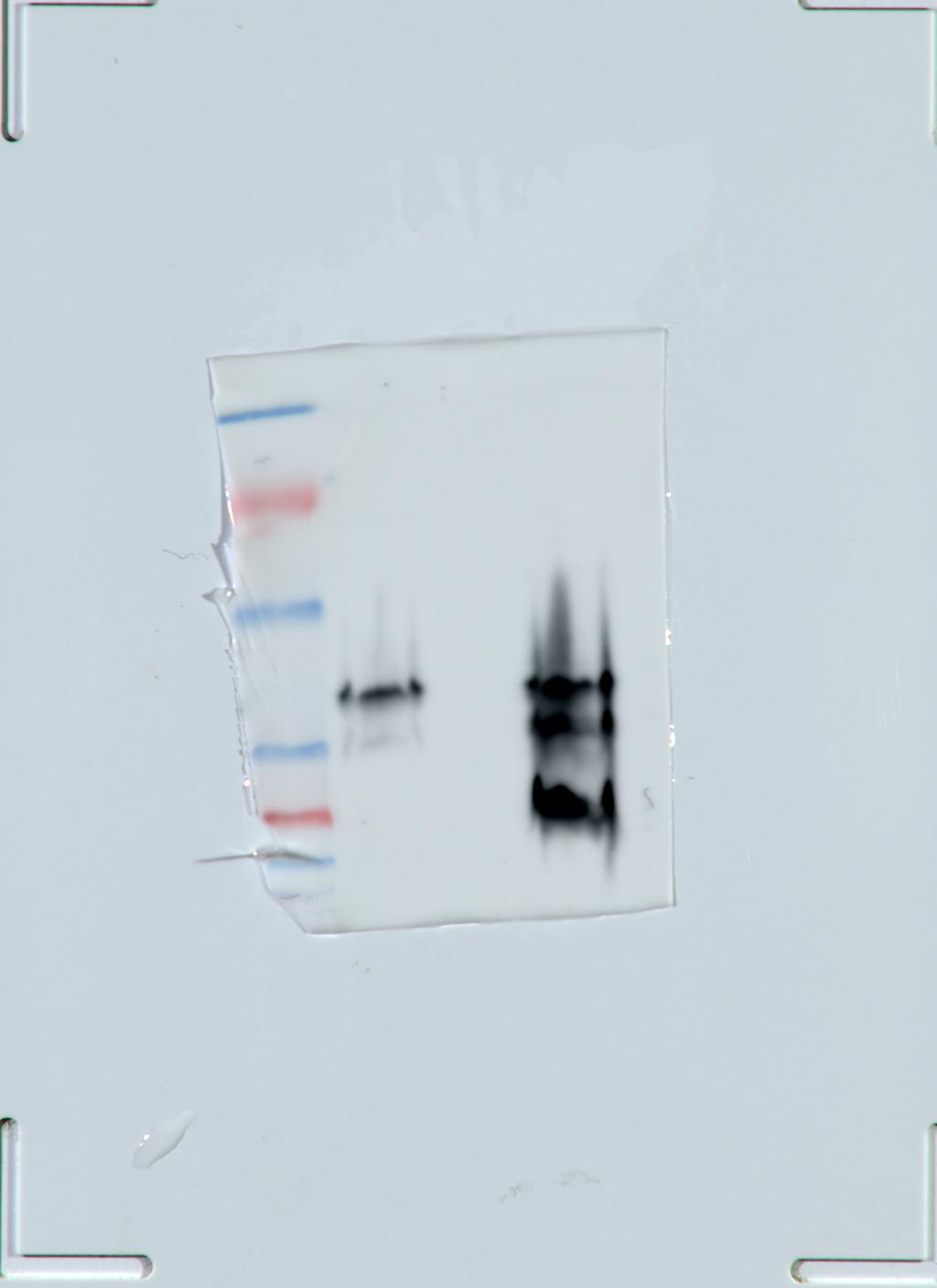

Supplement: Supplementary file 10 — Supplementary Material 10 [file 12896_2025_986_MOESM10_ESM.jpg]

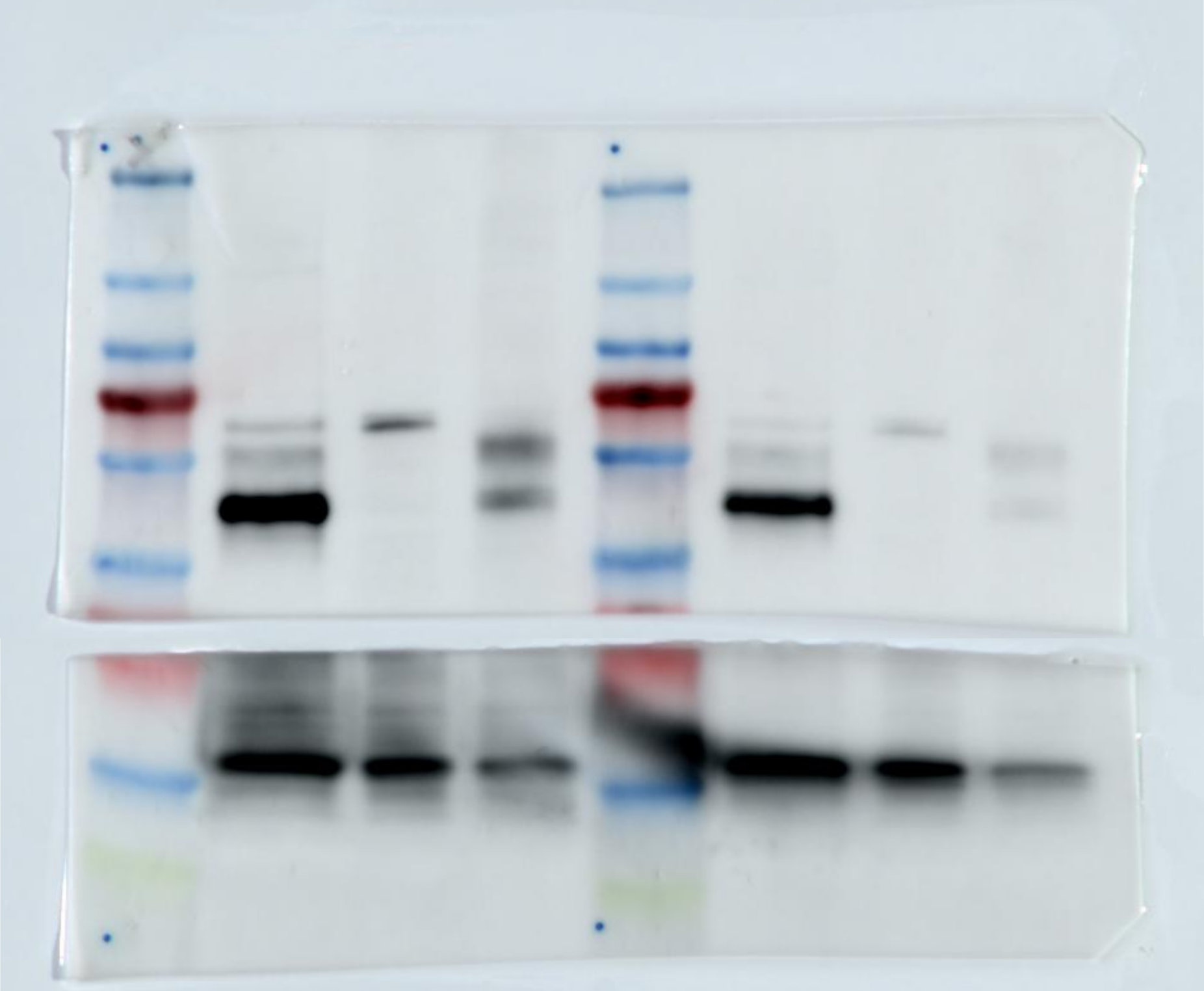

Supplement: Supplementary file 11 — Supplementary Material 11 [file 12896_2025_986_MOESM11_ESM.png]

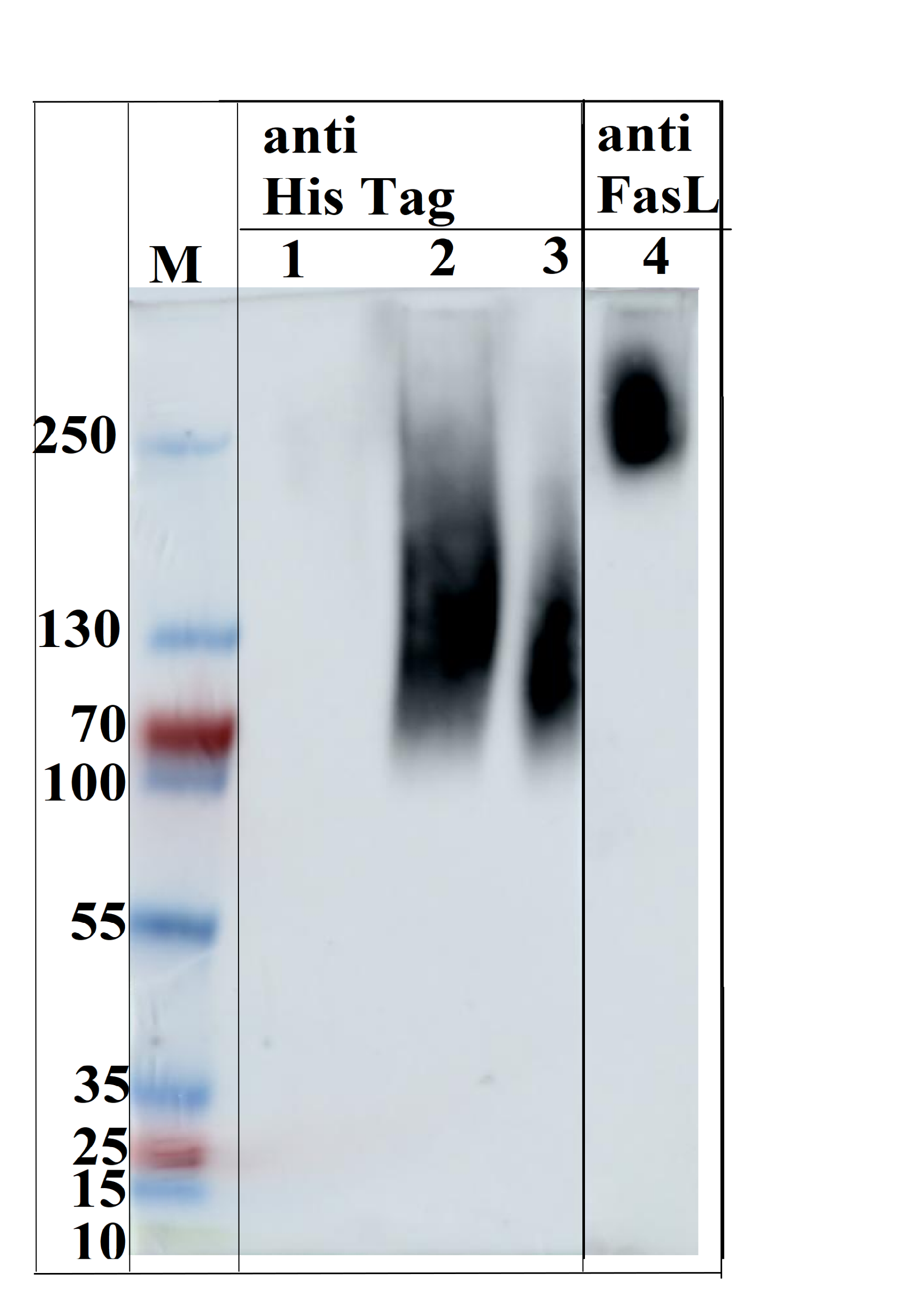

Supplement: Supplementary file 12 — Supplementary Material 12 [file 12896_2025_986_MOESM12_ESM.png]

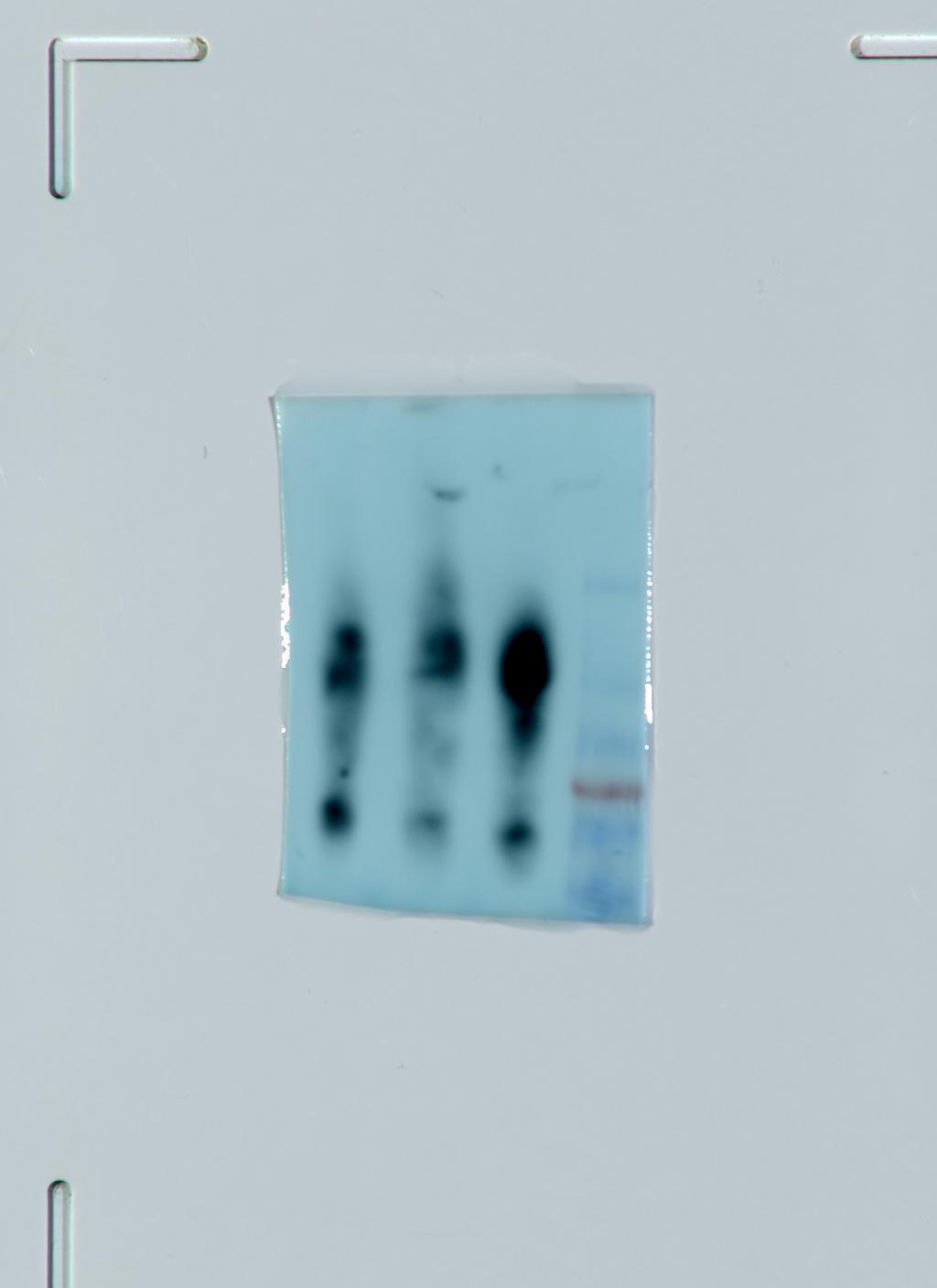

Supplement: Supplementary file 13 — Supplementary Material 13 [file 12896_2025_986_MOESM13_ESM.jpg]
