# Supplementary material for: High yield purification of an isoleucine zipper-modified CD95 ligand for efficient cell apoptosis initiation and with biotin or DNA-oligomer binding domain to probe ligand functionalization effects
Source: BMC Biotechnol. 2025 Jul 1;25:64. doi: 10.1186/s12896-025-00986-2 (PMC12219679; doi:10.1186/s12896-025-00986-2)
Supplement: Supplementary file 14 — Supplementary Material 14 [file 12896_2025_986_MOESM14_ESM.pdf]

Supplementary Information

High Yield Purification of an Isoleucine Zipper-Modified CD95 Ligand for Efficient Cell Apoptosis Initiation and with Biotin or DNA-oligomer Binding Domain to Probe Ligand Functionalization Effects

Xiaoyue Shang[1,2], Nina Bartels[1], Johann Moritz Weck[3], Sabine Suppmann[4], Jérôme Basquin[3], Gajen Thaventhiran[1], Amelie Heuer-Jungemann[3,5] and Cornelia Monzel[1,2]

1. Experimental Medical Physics, Heinrich Heine University, Universitätsstrasse 1, 40225 Düsseldorf, Germany

2. Present address: 2nd Institute of Physics, University of Stuttgart, Pfaffenwaldring 57, 70569 Stuttgart, Germany; E-mail: cornelia.monzel@pi2.uni-stuttgart.de

3. Max Planck Institute of Biochemistry, Am Klopferspitz 18, 82152 Martinsried, Germany

4. Fraunhofer Institute for Translational Medicine and Pharmacology ITMP, Nonnenwald 2, 82477 Penzberg, Germany

5. Present address: Hybrid Bionanosystems, TU Dortmund, Otto-Hahn-Str. 4a, 44227 Dortmund, Germany

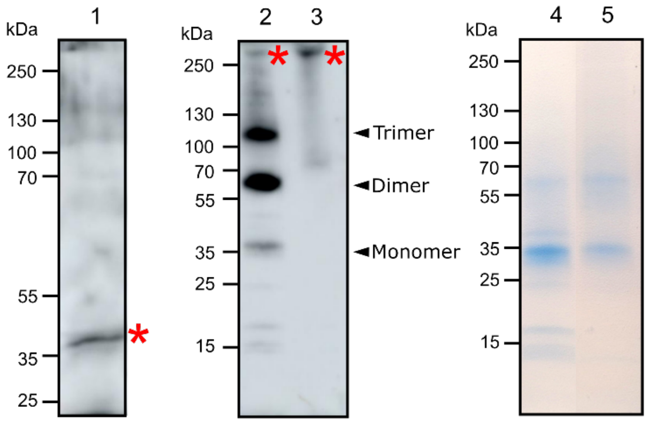

Figure S1. Lane 1: Detection of His-IZ-CD95L expression by western blot, using an anti-His-tag antibody. **Lane 2:** Western blot analysis of non-reduced His-IZ-CD95L, probed by anti-His-tag antibody. The quantification based on intensity ratios shows a trimer at 35.5 %, a dimer at 50 %, and a monomer at 14.5 % (ImageJ quantification). **Lane3:** His-IZ-CD95L cross-linked by 4 % formaldehyde (FA), reveals the presence of a high-order oligomer (indicated by asterisks). **Lane 4 and Lane 5:** Purity comparison of His-IZ-CD95L before and after size exclusion chromatography, with observed purities of 86 % and 97 %, respectively.

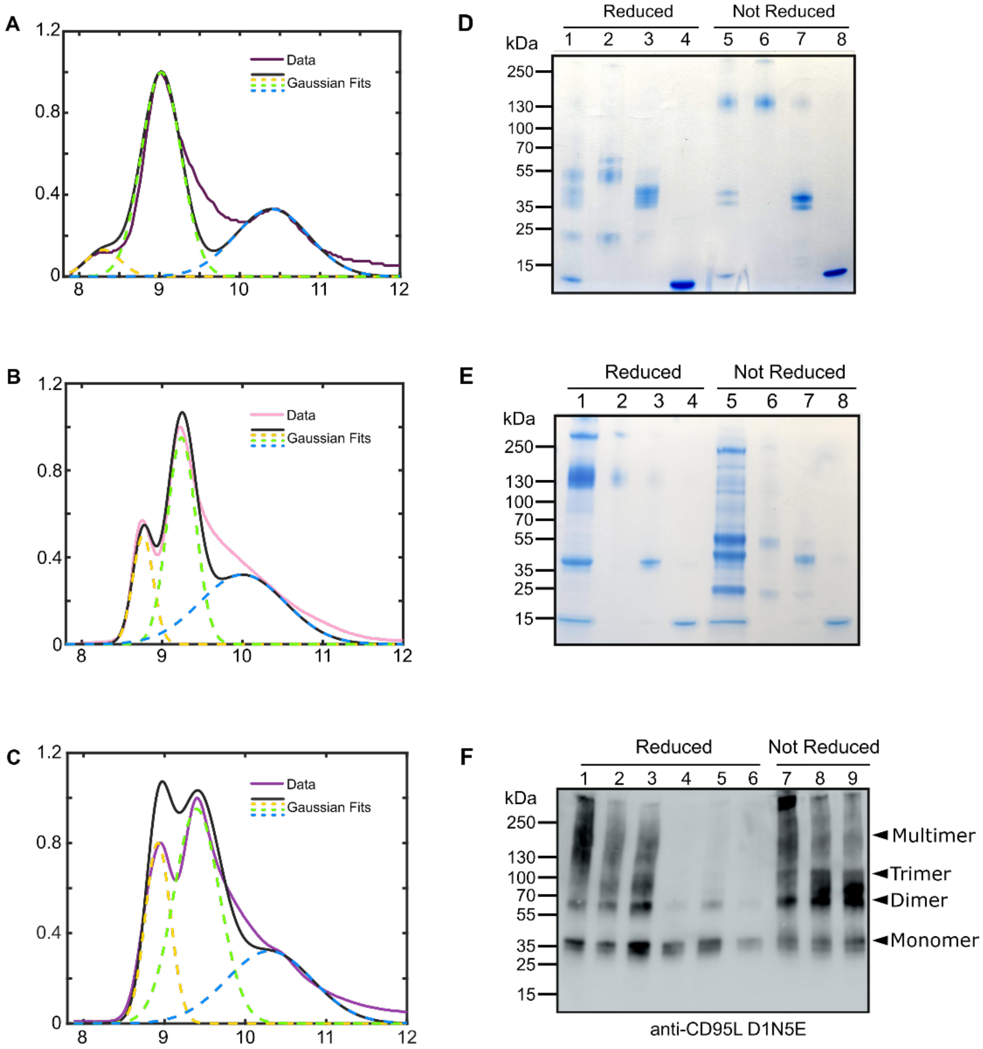

Figure S2. Size exclusion chromatogram of His-IZ-CD95L, IZ-CD95L, Biotin-IZ-CD95L, fitted with three Gaussian curves shown in **A B C** respectively with first, second, third Gaussian distribution shown as yellow, green, blue dashed lines. The percentage of 3 Gaussians can be calculated as 6 %, 58 %, and 36 % in **A**, 15 %, 55 %, 30 % in **B**, and 22 %, 48 %, 30 % in **C**. **D:** SDS-PAGE verification of protein standard (Sigma). Lane 1-4: reduced protein mix before SEC, fraction peaks of 150 kDa, 44 kDa, 14 kDa. Lane 5-8: non-reduced protein mix before SEC, fraction peaks of 150 kDa, 44 kDa, 14 kDa. **E:** SDS-PAGE verification of protein standard (Aglient). Lane 1-4: non-reduced protein mix before SEC, fraction peaks of 150 kDa, 45 kDa, 17 kDa. Lane 5-8: reduced protein mix before SEC, fraction peaks of 150 kDa, 45 kDa, 17 kDa. **F.** Western blot identification of Biotin-IZ-CD95L (lane 1-3, peak A-C) and IZ-CD95L (lane 4-6, peak A-C) under reduced conditions. Biotin-IZ-CD95L (lane 7-9, peak A-C) was tested under non-reduced conditions.

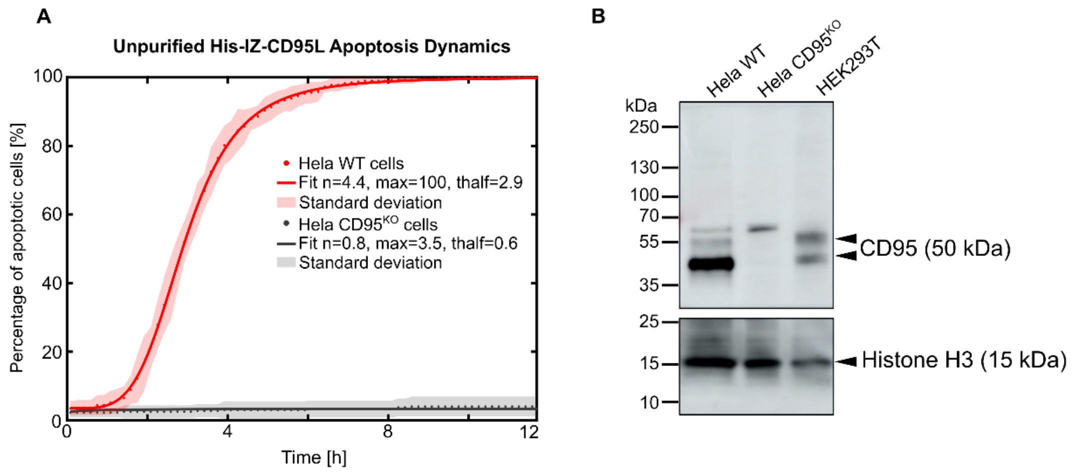

Figure S3. A: Functionality assay of His-IZ-CD95L before purification on Hela WT and Hela CD95^{KO} cell lines. A total of 300 µL of L15 complete medium, containing His-IZ-CD95L secreted after 3 days of transfection in HEK293T cells, was incubated overnight with the HeLa WT (shown in red) or Hela CD95^{KO} (shown in gray) cell line. Data points were fitted with the Hill equation with a result for Hela WT: the Hill coefficient (n) = 4.4, the maximum percentage of apoptotic cells (max) = 100 %, and the halflife-time (thalf) = 2.9 h; for Hela CD95^{KO}: the Hill coefficient (n) = 0.8, the maximum percentage of apoptotic cells (max) = 3.5 %, and the halflife-time (thalf) = 0.6 h. The graph reaches a value of 100 % apoptotic cells, which typically occurs at high ligand concentrations, here estimated for the unpurified His-IZ-CD95L to 2000 ng/mL. As a negative control, the Hela CD95^{KO} cell line showed negligible apoptosis events. **B:** Western blot test of native CD95 expression on Hela WT, Hela CD95^{KO}, and HEK293T cell line using anti-CD95 antibody (JJ0942). Equal amount protein loading was verified by anti-Histone H3 (Agerisa, AS10 710) antibody. Full length CD95 shows signal bands at 55 and 45 kDa. It can be observed that a variant of CD95 with high molecular weight was detected in HelaCD95^{KO} cell line, possibly coming from highly glycosylated cytosolic CD95, which is not properly inserted on plasma membrane. The Hela CD95^{KO} cell line was generated by CRISPR/CAS9 system and selected by CD95L incubation according to (1). Thus, cells expressing cytosolic CD95 were not killed and escaped from the screening step. However, this low amount expression of unfunctional CD95 does not interfere with apoptosis experiment, as shown in Figure S3A the gray curve with no observable cell death events. Besides, membrane CD95 quantification was determined with QIFIKIT in our previous study (2), where CD95 on Hela CD95^{KO} cell line was below the detection limit.

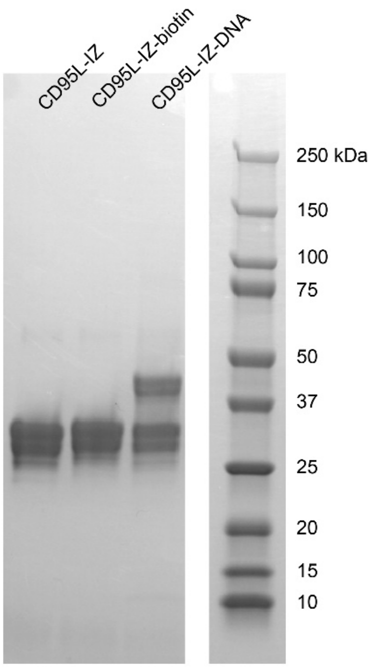

Figure S4. Denaturing SDS-PAGE gel of CD95L-IZ functionalized with biotin or DNA in comparison with non-functionalized CD95L-IZ.

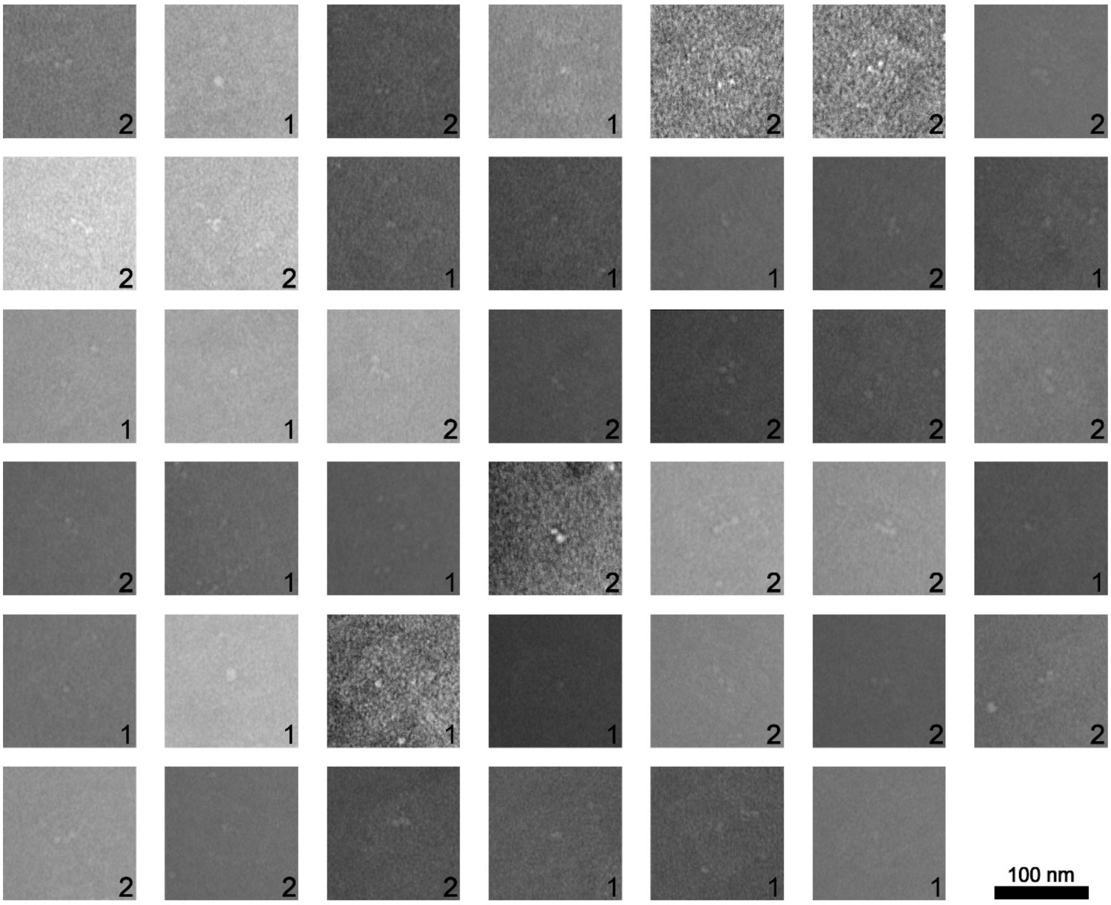

Figure S5. Montage of cropped TEM micrographs of DNA origami with CD95L attached to them. The number of FasL on the respective DNA origami is written in the lower right corner of every cropped image. For better visibility, the contrast was enhanced in some micrographs. The probability of CD95L attachment is ~ 70 %.

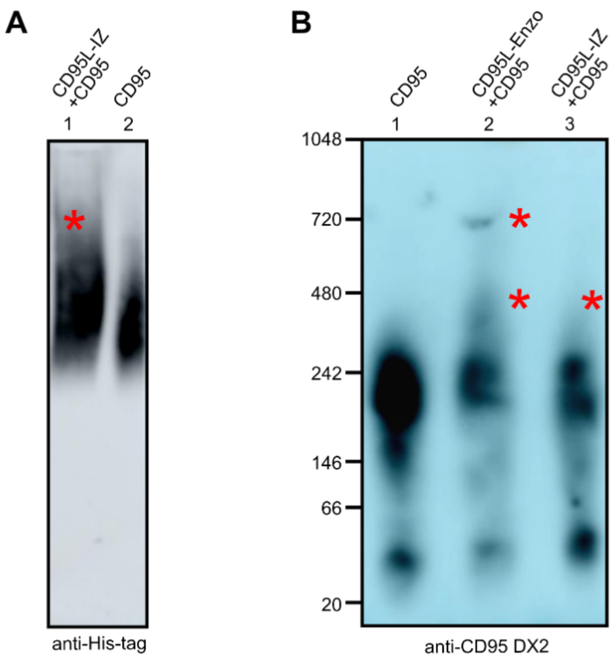

Figure S6. Native PAGE analysis of CD95L-IZ or CD95L-ENZO binding to CD95. A Clear native PAGE analysis of CD95L-IZ-biotin binding to CD95. Lane 1: 100 ng of CD95L-IZ-Biotin was incubated together with 100 ng CD95 (Sino Biological, His-tagged), and lane 2: 100 ng CD95 alone. The membrane was probed with an anti-His-tag antibody (Biolegend) to detect the shift in the CD95 signal band (indicated by an asterisk symbol). **B** Blue native page analysis of CD95L-IZ-biotin binding to CD95. The blot was probed with anti-CD95 antibody (Miltenyi Biotec, clone DX2), with lane 1: 100 ng CD95 alone (Sino Biological, His-tagged), lane 2: 100 ng CD95 incubated with 10x molar excess of CD95L-Enzo, and lane 3: 100 ng CD95 incubated with 10x molar excess of CD95L-IZ. Bands at higher molecular weight, indicated by asterisks, show fractions of CD95 forming complexes with CD95L-ENZO or CD95L-IZ. Unstained protein standard (Invitrogen) was used as molecular marker.

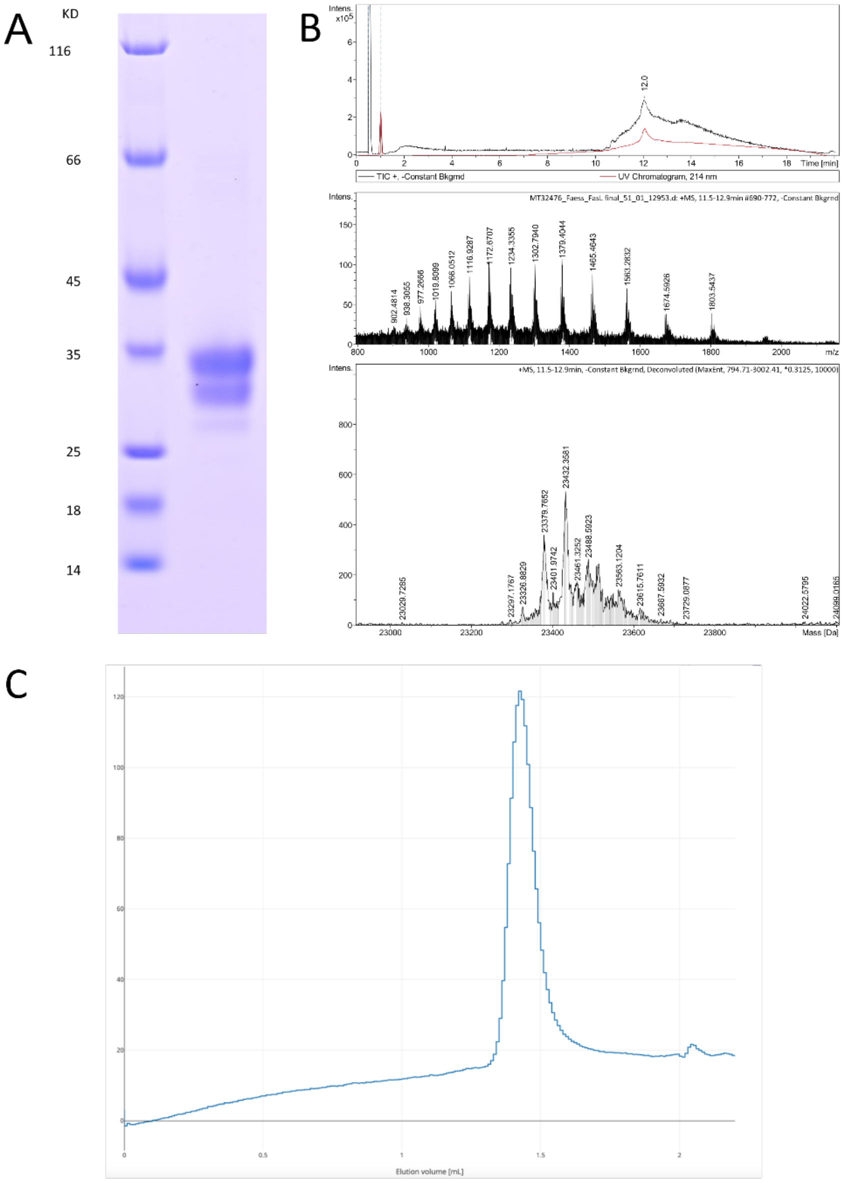

Figure S7. Quality control of IZ-CD95L produced from the stable cell line (method 2). **A.** SDS-PAGE analysis with gradient gel 4-12 %. Lane 1: molecular weight marker, Lane 2 purified IZ-CD95L. The purified protein runs as a double band reflecting different glycosylation states. **B.** Liquid chromatography-mass spectrometry (LC-MS) analysis with the intact mass of the purified batch. The mass spectrometry spectra confirm different glycosylation states. **C.** Analytical gel filtration with a Superdex™ 200 increase 3.2/300 GL column in a buffer containing 20 mM Hepes (N-2-hydroxyethylpiperazine-N'-2-ethanesulfonic acid) pH 7.5, 150 mM NaCl, and 2 mM DTT.

Table S1. Significance values of one-way ANOVA (analysis of variance) test for the data given in Figure 4, with * $p < 0.05$, ** $p < 0.01$, *** $p < 0.001$.

A: HelaWT	Test components	Degree of freedom	F ratio	P value
1	Lanes 1-14	F(13, 26)	22.936	1.6070e-11
2	Lanes 1-4	F(3, 6)	17.809	0.0007
3	Lanes 5-7	F(2, 4)	5.068	0.0514
4	Lanes 12-14	F(2, 4)	6.557	0.0309
5	Lanes 8-11	F(3, 6)	2.488	0.1346
6	Lanes 1-7	F(6, 12)	13.190	4.7082e-05
7	Lanes 8-14	F(6, 12)	7.375	0.0010
8	Lanes 1-11	F(10, 20)	28.087	3.0784e-10
9	Lanes 5-14	F(9, 18)	18.274	8.5428e-08
10	Lanes 5-11	F(6, 12)	30.133	3.1314e-07

B: HEK293	Test components	Degree of freedom	F ratio	P value
1	Lanes 1-6	F(5, 10)	0.187	0.9620
2	Lanes 5-6	F(1, 2)	0.011	0.9203
3	Lanes 1-5	F(4, 8)	0.115	0.9742

C:MCF7	Test components	Degree of freedom	F ratio	P value
1	Lanes 1-11	F(10, 20)	3.679	0.0052
2	Lanes 7-11	F(4, 8)	0.947	0.4761
3	Lanes 1-6	F(5, 10)	0.714	0.6245
4	Lanes 1-5	F(4, 8)	0.810	0.5464
5	Lanes 1-4	F(3, 6)	0.988	0.4458
6	Lanes 1-3	F(2, 4)	1.007	0.4197
7	Lanes 4-6	F(2, 4)	0.214	0.8128
8	Lanes 3-5	F(2, 4)	0.166	0.8507

D: SEC	Test components	Degree of freedom	F ratio	P value
1	Lanes 1-4	F(3, 6)	0.135	0.9361

References

1. Liesche C, Venkatraman L, Aschenbrenner S, Grosse S, Grimm D, Eils R, et al. Death receptor-based enrichment of Cas9-expressing cells. BMC Biotechnol. 2016 Feb 16;16:17.
2. Bartels N, M van der Voort NT, Opanasyuk O, Felekyan S, Greife A, Shang X, et al. Advanced multiparametric image spectroscopy and super-resolution microscopy reveal a minimal model of CD95 signal initiation. Sci Adv [Internet]. 2024;10(35):eadn3238. Available from: https://www.science.org
